# 3D Topological Inorganic Electrides: Screening, Properties, and Applications

**DOI:** 10.1002/advs.202507469

**Published:** 2025-07-03

**Authors:** Zhenzhou Guo, Weizhen Meng, Qianwen Zhang, Xia Cheng, Shiyao Wang, Yalong Jiao, Xiaoming Zhang, Ying Liu, Tie Yang

**Affiliations:** ^1^ College of Physics Hebei Key Laboratory of Photophysics Research and Application Hebei Normal University Shijiazhuang 050024 China; ^2^ School of Physical Science and Technology Southwest University Chongqing 400715 China; ^3^ State Key Laboratory of Reliability and Intelligence of Electrical Equipment, and School of Materials Science and Engineering Hebei University of Technology Tianjin 300130 China; ^4^ MDX Research Center for Element Strategy Institute of Integrated Research Institute of Science Tokyo Yokohama 226‐8503 Japan; ^5^ Institute for Superconducting and Electronic Materials (ISEM) Faculty of Engineering and Information Sciences University of Wollongong Wollongong New South Wales 2500 Australia

**Keywords:** 3D inorganic electrides, interstitial anion electrons, NH_3_ synthesis, topological states, work function

## Abstract

The convergence of inorganic electrides and topological quantum phenomena has ushered in novel paradigms for designing advanced quantum materials. While low‐dimensional (0–2D) topological inorganic electrides have garnered considerable attention, their three‐dimensional (3D) counterparts—featuring intricate interstitial electron networks—remain entirely uncharted territory. Herein, the identification of twelve 3D inorganic electrides within the rare‐earth hydride family is reported, nine of which are previously unreported. First‐principles calculations reveal a diverse magnetic landscape: two systems exhibit ferromagnetic ordering, while seven demonstrate antiferromagnetic configurations. Strikingly, these materials host rich topological states, encompassing nodal points, nodal lines, and associated surface signatures such as Fermi arcs and drumhead‐like states. When spin‐orbit coupling is introduced, the magnetic ordering breaks time‐reversal symmetry, thereby generating substantial Berry curvature and resulting in a relatively large anomalous Hall conductivity (939 S cm^−1^). Furthermore, these inorganic electrides exhibit ultralow work functions (2.6–3.9 eV) on rare‐earth‐terminated surfaces. Under external electric fields, the 3D interstitial electrons migrate to the surface, forming a quasi‐2D electron gas. Significantly, such low work functions can effectively activate N_2_, enhancing catalytic NH_3_ synthesis. These findings establish an ideal platform to explore 3D inorganic electrides, along with their topological features, anomalous transport phenomena, low work functions, and NH_3_ synthesis.

## Graphical abstract

In inorganic electrides, zero‐, one‐, and two‐dimensional (0D, 1D, and 2D) interstitial anionic electrons interconnect within lattice cavities, collectively forming a three‐dimensional (3D) network, further coupling magnetism and topological features, leading to the emergence of 3D topological inorganic electrides. Furthermore, rare‐earth‐based hydrides (ReH_2_), as promising candidates, exhibit potential applications in electron emission devices and NH_3_ synthesis.

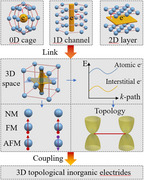

## 1 Introduction

Over the past decade, the synergistic integration of group theory analysis, high‐throughput screening, chemical rules, and advanced experimental characterization has led to the successful prediction and verification of a wide range of topological materials.^[^ ^1^, ^2^, ^3^, ^4^, ^5^, ^6^ ^]^ Among these, topological semimetals have emerged as a particularly rich class, hosting various types of topological fermions, including 0D nodal points (NPs), 1D nodal lines (NLs), and 2D nodal surfaces (NSs).^[^ ^7^, ^8^, ^9^, ^10^ ^]^ Remarkably, certain fermions exhibit distinct surface signatures, such as Fermi arcs and drumhead‐like states. These topologically protected surface states facilitate both dissipationless electron transport and high carrier mobility on solid surfaces.^[^ ^11^, ^12^ ^]^ Furthermore, their unique surface electronic structures generate abundant highly active sites for catalytic reactions.^[^ ^13^, ^14^, ^15^ ^]^ Intriguingly, the introduction of magnetic ordering breaks time‐reversal (*T*) symmetry, which may induce unconventional transport phenomena under spin‐orbit coupling (SOC), such as the quantum anomalous Hall effect.^[^ ^16^, ^17^, ^18^ ^]^ Recently, topological quantum chemistry theory has demonstrated that the emergence of these topological states originates from electron states far from the atomic orbital limit.^[^ ^19^ ^]^ This mechanism is intrinsically linked to band inversion induced by non‐bonding interstitial anionic electrons (IAEs) in inorganic electrides.

Unlike conventional ionic, covalent, and intermetallic compounds, inorganic electrides feature a positively charged atomic framework hosting IAEs.^[^ ^20^, ^21^, ^22^, ^23^ ^]^ The spatial distribution of IAEs, governed by the cavity size effect in the crystal structure, occurs in four distinct configurations: 0D cages, 1D channels, 2D interlayers, and 3D spaces. Remarkably, the quasi‐free nature of IAEs endows inorganic electrides with a range of exceptional properties, including low work function (WF), enhanced catalytic activity, high electron density, unique magnetic behavior, abundant topological states, and pressure‐induced superconductivity.^[^ ^21^, ^22^, ^23^, ^24^, ^25^, ^26^, ^27^ ^]^ Particularly noteworthy is the emerging field of topological inorganic electrides (TIEs), where the coupling between nontrivial band topology and IAEs has become a focal point in condensed matter physics. Subsequently, numerous TIEs have been confirmed by theory and experiment, such as six‐fold excitations arising from 0D IAE in Ca_6_Al_7_O_16_/Li_12_Mg_3_Si_4_, NSs generated by 1D IAE in Sr_3_CrN_3_, and NLs formed by 2D IAE in ferromagnetic (FM) Gd_2_C.^[^ ^28^, ^29^, ^30^, ^31^, ^32^, ^33^, ^34^ ^]^ The coupling between inorganic electrides and topological properties has opened new horizons for exploring multifunctionality in single‐material systems. However, to the best of our knowledge, research on topological states has been limited to 0D,1D, and 2D inorganic electrides, and the design of 3D TIEs remains a completely unexplored field.

In this work, we identified twelve 3D inorganic electrides, namely rare earth hydrides (ReH_2_), among which two displayed FM ordering and seven exhibited antiferromagnetic (AFM) ordering. Taking the FM 3D inorganic electride CeH_2_ as an example, it can simultaneously host three distinct spin topological states: a quadratic triple‐degenerate point, a closed Weyl NL, and two open Weyl NLs. Furthermore, Fermi arc and drumhead‐like surface states originating from NPs and NLs were clearly observed. When including the SOC effect, the FM‐induced *T*‐symmetry breaking generates significant Berry curvature near the Fermi level, manifesting as a high anomalous Hall conductivity (σ_yz_ = 939 S cm^−1^). Notably, these 3D inorganic electrides exhibit significantly low WFs (Φ_WF_ <4 eV) on the (001) surface. Under the control of an external electric field along the *k*_z_ direction, the 3D IAEs can be effectively extracted from their interstitial positions into the vacuum layer, forming an approximate 2D electron gas (2DEG). Furthermore, the strong electron‐donating nature of inorganic electrides can significantly lower the activation barrier for N_2_ dissociation, thereby efficiently enhancing the NH_3_ synthesis process. These findings provide a potential platform for exploring 3D inorganic electrides, topological properties, magnetism, and related applications.

## 2 Designing 3D Topological Inorganic Electrides

**Figure 1:**
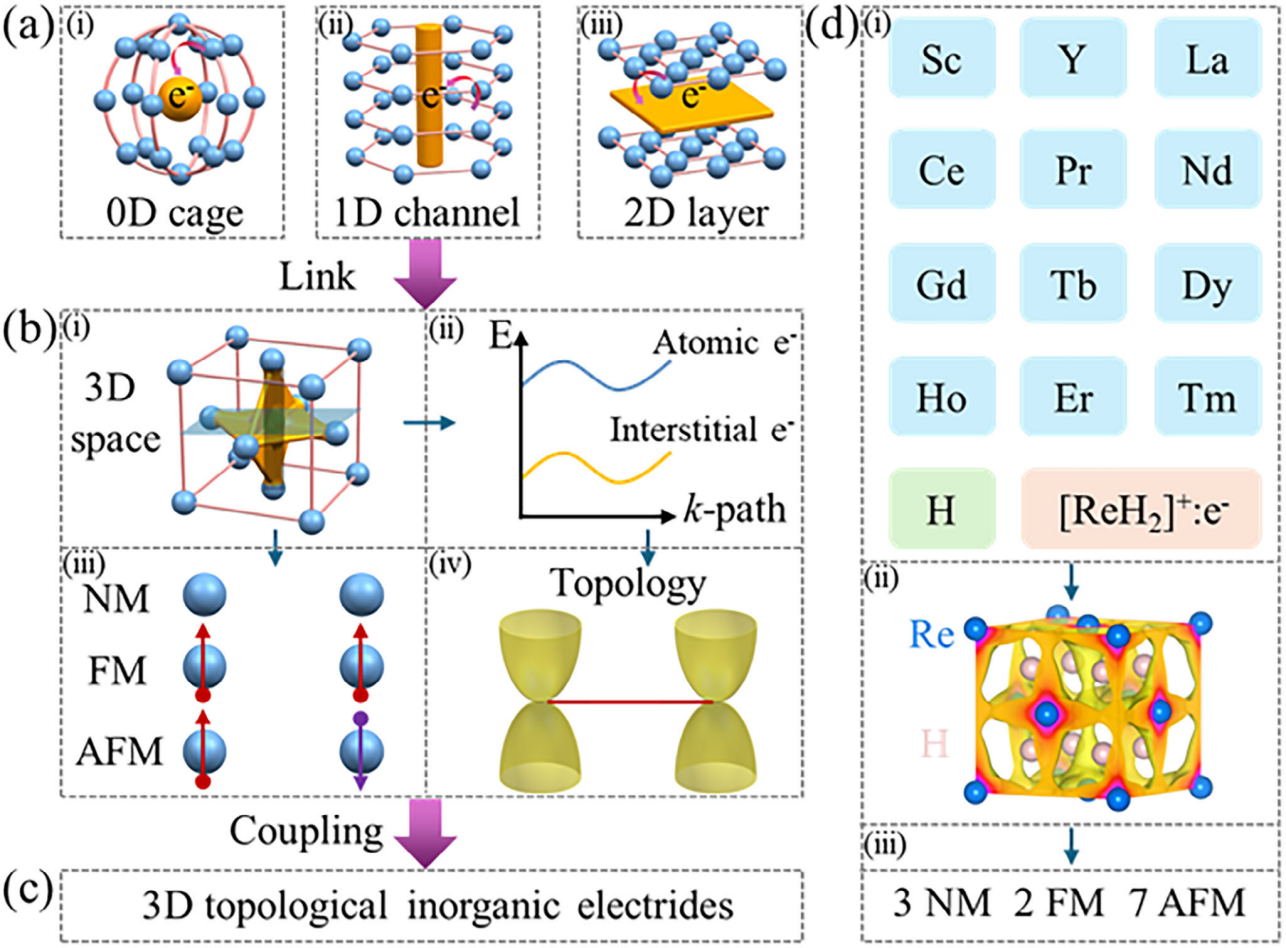
a) shows that the IAEs (e^−^) are localized within (i) 0, (ii) 1, and (iii) 2D lattice cavities of inorganic electrides. b)‐(i) Interconnection of 0D, 1D, and 2D IAEs into a 3D morphology. (ii) Magnetic classification of 3D inorganic electrides. (iii) Schematic diagram of the electronic band structure for 3D inorganic electrides. (iv) Topological states induced by interstitial e^−^ in 3D inorganic electrides. c) Formation of 3D topological inorganic electrides through coupling of 3D inorganic electrides with topological properties. d)‐(i) Rare‐earth‐based hydrides (ReH_2_). (ii) Partial charge density (PCD) below the Fermi level (−0.5 to 0 eV) of ReH_2_. (iii) Magnetic properties of twelve candidates: three NM, two FM ordering, and seven AFM ordering.

In inorganic electrides, the dimensionality of IAEs is primarily governed by the geometric framework formed by cations. To date, most screened inorganic electrides predominantly exhibit three fundamental configurations: cages, channels, and interlayers, corresponding to 0, 1, and 2D inorganic electrides, as shown in **Figure** **1**a. Notably, these confined IAEs can further interconnect within their host lattice cavities, collectively forming extended 3D morphologies (see Figure 1b‐(i)). In other words, 3D IAEs can simultaneously accommodate 0D, 1D, and 2D IAEs. Moreover, certain inorganic electrides containing transition metals or rare‐earth elements can exhibit magnetic behavior owing to unpaired electrons in their *d‐/f*‐orbitals. This enables the classification of inorganic electrides into three distinct types of magnetic ordering: FM, AFM, and non‐magnetic (NM), as shown in Figure 1b‐(ii). The electronic band structures near the Fermi level in inorganic electrides typically exhibit hybrid contributions from both atomic orbitals and interstitial electrons. When their emergent topological states near the Fermi level demonstrate significant participation from IAEs, such systems can be regarded as TIEs (see Figure 1b‐(iii,iv); Figure S1, Supporting Information). Therefore, when a solid simultaneously satisfies the following two conditions: i) the presence of 3D IAEs in lattice cavities; ii) the existence of topological states near the Fermi level with significant contributions from these IAEs, it can be defined as a 3D TIE (see Figure 1c).

Mizoguchi et al. identified LnX_2_ (Ln = La, Ce, Y) as prototypical 3D inorganic electrides.^[^ ^35^, ^36^ ^]^ Building upon this foundation, we focused on rare‐earth‐based hydrides, leading to the discovery of nine novel 3D inorganic electrides, namely ReH_2_ (Re = Sc, Pr, Nd, Gd, Tb, Dy, Ho, Er, Tm), as shown in Figure 1d‐(i). Significantly, all these materials have been experimentally synthesized (see Section S2, Supporting Information). Electronegativity analysis reveals that Re exhibits lower electronegativity than H, indicating that the outermost *d‐/f*‐orbital electrons of Re in ReH_2_ are more easily transferred to the *s*‐orbital of the H atom (see Table S1, Supporting Information). Valence state analysis demonstrates that Re predominantly adopts a +3 oxidation state (Re^3+^), while H atoms gain one electron each to form H^−^, confirming [ReH_2_]^+^: e^−^ as a class of electron‐rich materials. Remarkably, these geometric cavities (V = 20–24 Å^3^) formed by Re atoms significantly exceed the spatial constraints of conventional 0D‐2D inorganic electrides. Partial charge density (PCD) calculations further reveal that the excess electrons are transferred to the lattice cavities formed by Re atoms, exhibiting a 3D morphology (see Figure 1d‐(ii)). Furthermore, energy calculations of different magnetic configurations demonstrate that Sc/Y/LaH_2_, Ce/NdH_2_, and Pr/Gd/Tb/Tm/Dy/Ho/ErH_2_ exhibit NM, FM, and AFM behaviors, respectively (see Figure 1d‐(iii); Table S2, Supporting Information). Next, we will systematically investigate their electronic structures, topological features, transport properties, WF, and NH_3_ synthesis.

## 3 Example of 3D TIEs: FM Metal CeH_2_

### 3.1 Magnetism and Electronic Band Structure of CeH_2_

**Figure 2:**
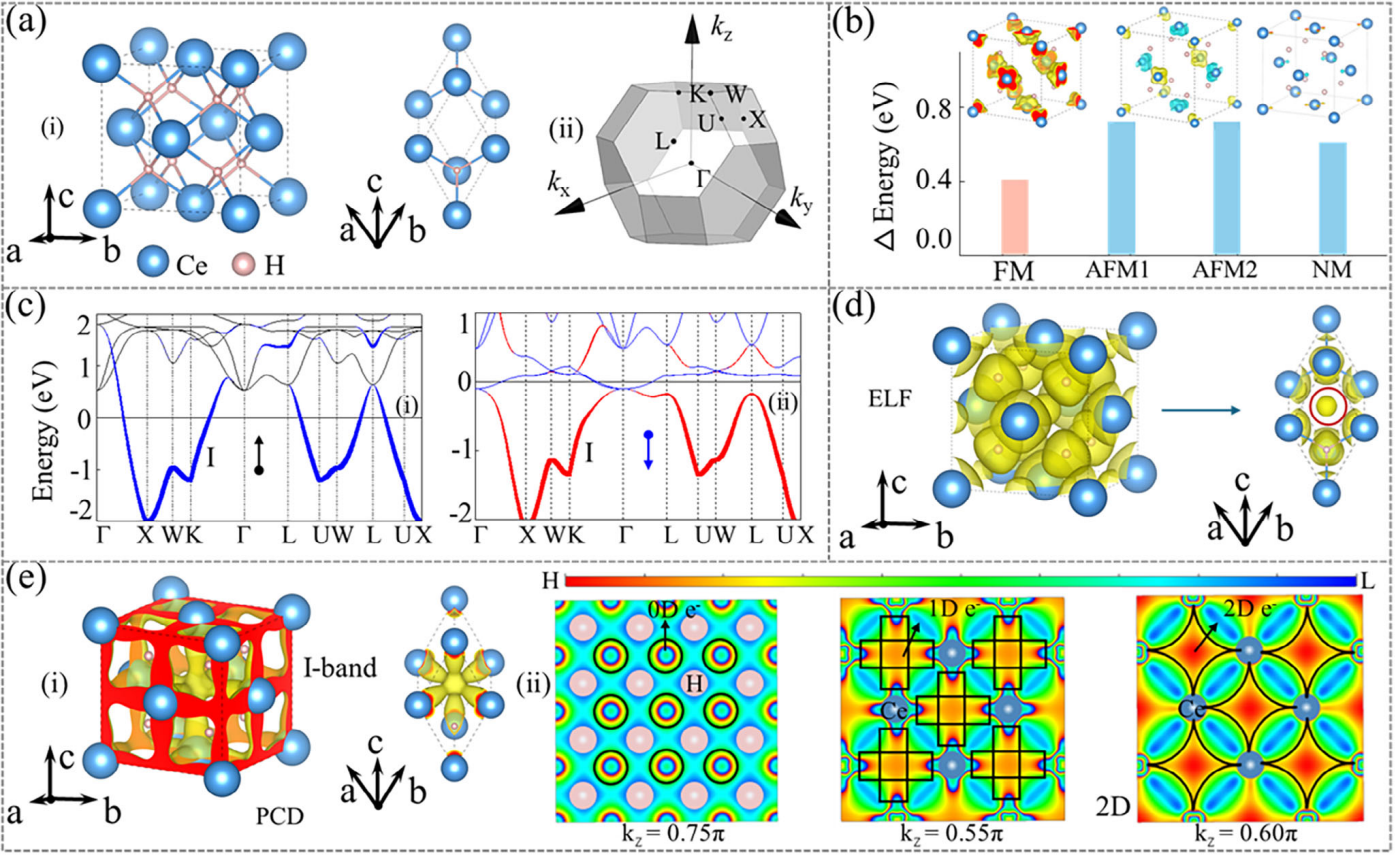
a)‐(i) Unit cell and primitive cell of CeH_2_. (ii) Brillouin zone (BZ) and high‐symmetry points of CeH_2_. () shows the difference between the energies of different magnetic configurations and 48.5 eV, where the insets represent spin density maps of CeH_2_. c) Spin‐resolved electronic band structures: (i) spin‐up and (ii) spin‐down channels, where the blue and red bands represent the contributions of 3D IAEs. d) Electron localization function (ELF) maps of CeH_2_. e)‐(i) Partial charge density (PCD) maps of CeH_2_. (ii) 2D views of PCD at k_z_ = 0.75, 0.55, and 0.60*π* planes, respectively.

**Figure** **2**a shows the crystal structure and Brillouin zone (BZ) of CeH_2_, which belongs to space group 225 (*Fm‐3m*) with inversion symmetry (*P*). The unit cell of CeH_2_ consists of 4 Ce atoms and 8 H atoms (see Figure 2a‐(i)). The face‐centered Ce atoms form a regular octahedron (d_Ce‐Ce_ = 3.9 Å), inside which a cubic structure composed of H atoms is embedded (V_H_ = 20.8 Å^3^). This distinctive atomic arrangement creates a lattice cavity that facilitates the formation of 3D IAEs. In addition, the atomic positions and lattice parameters of the ReH_2_ primitive cell are shown in Tables S3 and S4 (Supporting Information). We then performed DFT+*U* calculations with distinct Hubbard *U* values applied to the *4f*‐orbitals of Ce atoms (*U*_Ce_ = 0, 3, 4, 5 eV), considering three magnetic configurations (FM, AFM1, and AFM2) (see Figure 2b; Figures S2 and S3, Supporting Information). These results consistently demonstrate that the FM ordering is the most stable ground state, with the observed magnetic moment localized on Ce atoms (0.698 µ_B_/Ce atom). The spin density maps corresponding to the most stable magnetic ground states for other candidates are provided in Figure S4 (Supporting Information).

In the FM ground state, CeH_2_ exhibits metallic characteristics in both spin channels (see Figure 2c). We can find that the IAEs primarily contribute to the I‐band located below the Fermi level. Through electron localization function (ELF) calculations, we observed the 0D interstitial electrons located at the (0.5, 0.5, 0.5) Wyckoff position in CeH_2_ (see Figure 2d). Remarkably, while ELF serves as a valuable indicator for identifying inorganic electrides,^[^ ^32^, ^33^, ^34^, ^37^ ^]^ it should not be regarded as the definitive criterion for determining the dimensionality of interstitial electrons—the primary evaluation should be based on PCD.^[^ ^38^, ^39^ ^]^ Subsequently, the PCD distribution of the I‐band is shown in Figure 2e‐(i), clearly demonstrating that the IAEs display a 3D morphology within the cubic framework formed by H atoms. Furthermore, these 3D IAEs can be separated into 0, 1, and 2D IAEs along the *k*_z_ direction (see Figure 2e‐(ii)). The electronic band structures, ELF, and PCD maps of other candidates are shown in Figures S5–S7 (Supporting Information).

### 3.2 Topological Properties of CeH_2_

**Figure 3:**
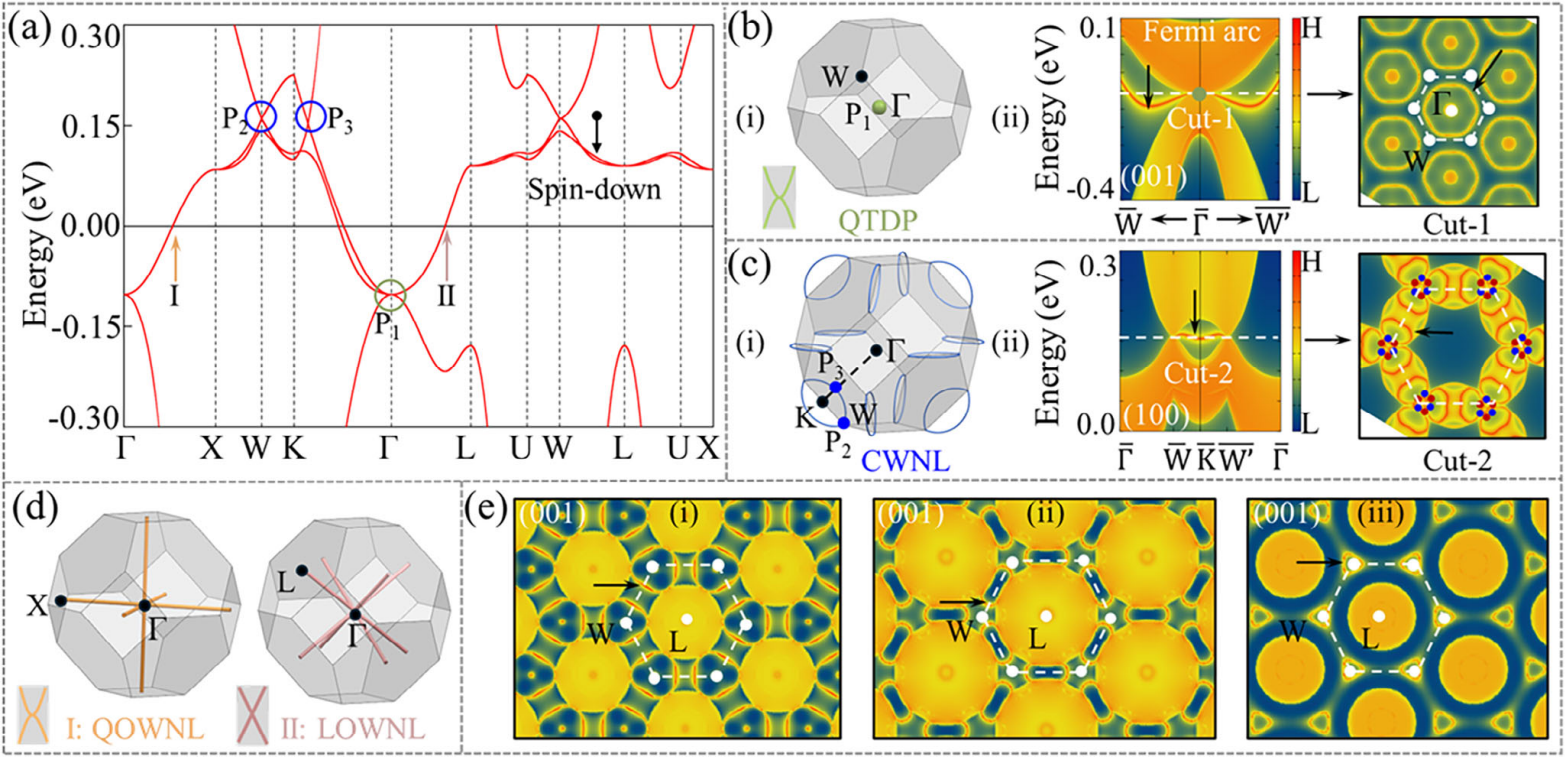
a) illustrates the electronic band structures under the spin‐down channel in CeH_2_. b)‐(i) depicts the position of QTDP in the BZ. (ii) shows the (001) surface spectrum of QTDP and the energy slice at the Cut‐1 position. c)‐(i) shows the position of CWNL in the BZ. (ii) shows the (100) surface spectrum of CWNL and the energy slice at the Cut‐2 position. d) shows the positions of QOWNLs in the BZ. e) shows the (001) surface energy slice of LOWNLs.

**Figure** **3**a displays the spin‐down electronic band structure of CeH_2_, highlighting three distinct band‐crossing features near the Fermi level: (i) a crossing point (P_1_) located at the high‐symmetry Γ point, (ii) another crossing (P_2_) occurring at the W point, and (iii) the third crossing (P_3_) along the Γ‐K path. In addition, bands I and II exhibit two degenerate lines along the Γ‐X and Γ‐L paths, respectively. Notably, the *T* symmetry can be preserved in the FM systems when only one spin channel is considered.^[^ ^40^, ^41^ ^]^ Symmetry analysis indicates the following key features: i) P_1_, with an irreducible representation (*IR*) of $\Gamma_{4}^{-}$, is a quadratic three‐fold degenerate point (QTDP) protected by $S_{6}^{-}$, **σ**_**x**_, **σ**_**z**_, *C*_2*c*_, and *T* symmetries (see Figure 3b‐(i)); ii) P_2_ and P_3_ form a closed Weyl nodal loop (CWNL) centered around the K‐point (see Figure 3c‐(i); Figure S9, Supporting Information); and iii) the degenerate band I, with an *IR* of *DT_5_*, corresponds to a quadratic open Weyl nodal line (QOWNL) with a zero Berry phase, while the degenerate band II, with an *IR* of *LD_3_*, exhibits a linear open Weyl nodal line (LOWNL) with a *π* Berry phase, which are protected by $C_{4y}^{+}$, σ_*x*_, *PT* (along the Γ‐X path) and $C_{3}^{+}$, σ_*db*_, *PT* (along the Γ‐L path) symmetries, respectively (see Figure 3d).

Remarkably, the (001) and (100) surface spectra exhibit three distinct characteristics: i) a fragile Fermi arc with pronounced hexagonal warping emerging from the QTDP (see Figure 3b‐(ii)), ii) a drumhead‐like surface state with three‐fold petal‐like features associated with the CWNL (see Figure 3c‐(ii)), and iii) a robust drumhead‐like surface state featuring triangular warping linked to the LOWNL (see Figure 3e). The coupling between surface electride states and topological surface states has potential applications in spin‐polarized transport,^[^ ^42^ ^]^ quantum devices,^[^ ^43^ ^]^ optoelectronic devices,^[^ ^30^ ^]^ topological field‐effect transistors,^[^ ^44^ ^]^ etc.

### 3.3 Anomalous Transport Properties of CeH_2_

**Figure 4:**
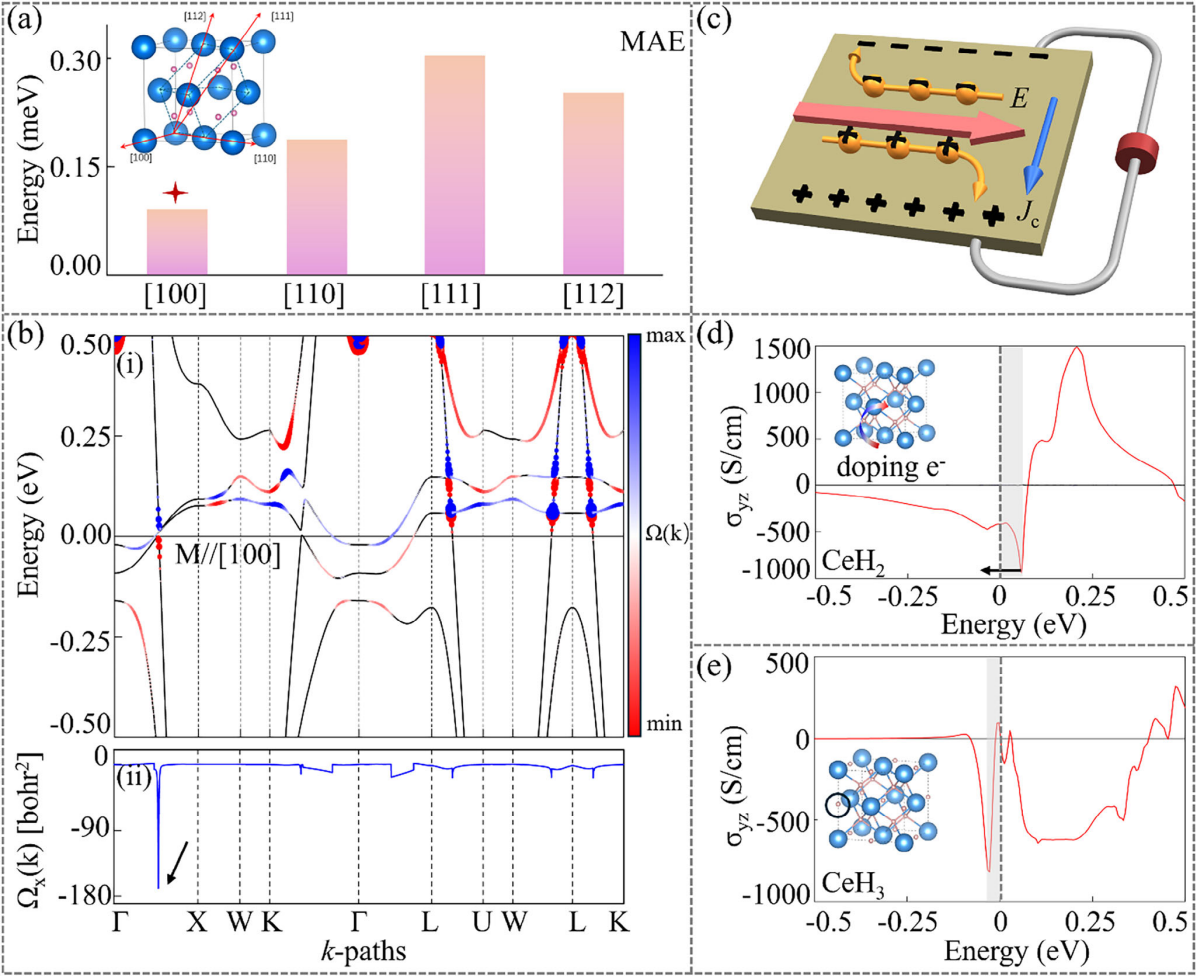
a) illustrates the MAE along different crystallographic directions for CeH_2_. b)‐(i) depicts the projected Berry curvature distribution for the electronic band with SOC along the [100] direction. (ii) shows the Berry curvature corresponding to the electronic structure in (i). c) Schematic diagram of the formation of anomalous Hall current in CeH_2_. d) shows the anomalous Hall conductivity of CeH_2_. e) illustrates the anomalous Hall conductivity of CeH_3_.

**Figure** **4**a shows the magnetic anisotropy energy (MAE) along four crystallographic directions: [100], [110], [111], and [112]. Our calculations indicate that the easy magnetization axis of CeH_2_ lies along the [100] direction. Figure 4b–(i) shows the projected Berry curvature distribution for the electronic band with magnetization aligned along the [100] crystallographic direction. The color‐coded plot reveals distinct regions of positive (red) and negative (blue) Berry curvature. Notably, when the SOC effect is included, these topological features are annihilated, resulting in the opening of distinct bandgaps at the original crossing points. This SOC‐induced band splitting generates significant Berry curvature, reaching a maximum value of 169.7 bohr^2^ along the Γ‐X path, as shown in Figure 4b–(ii). The momentum‐space Berry curvature distribution, originating from the breaking of T‐symmetry in the electronic structure, fundamentally determines the strength of the intrinsic anomalous Hall conductivity. In FM CeH_2_, this leads to the emergence of a transverse Hall current, even without an external magnetic field (see Figure 4c).

The calculations indicate a substantial intrinsic anomalous Hall conductivity of 414.6 S cm^−1^ at the Fermi level (E_F_ = 0 eV) (see Figure 4d). Notably, slightly above the Fermi level, multiple originally degenerate NPs are split by the SOC, resulting in enhanced Berry curvature (see Figure 4b‐(ii)). Consequently, the anomalous Hall conductivity exhibits a pronounced peak of ≈939 S cm^−1^ at 0.06 eV. Thus, electron doping of 0.06 e^−^/unit cell should substantially boost the anomalous Hall conductivity. Indeed, this regulatory mechanism can be achieved by doping with H atoms. Using experimentally synthesized CeH_3_ as an example,^[^ ^45^ ^]^ the calculations demonstrate that CeH_3_ still maintains the FM ground state with the [100] easy magnetization axis (see Figure S10, Supporting Information). Remarkably, the anomalous Hall conductivity peak of 840 S cm^−1^ shifts to 0.03 eV below the Fermi level (see Figure 4e). Moreover, we calculated the anomalous Hall conductivities for the [110], [111], and [112] magnetization directions, yielding values of 449.5, 330.3, and 309.2 S cm^−1^, respectively (see Figure S11, Supporting Information).

## 4 WFs and NH_3_ Synthesis of 3D TIEs

**Figure 5:**
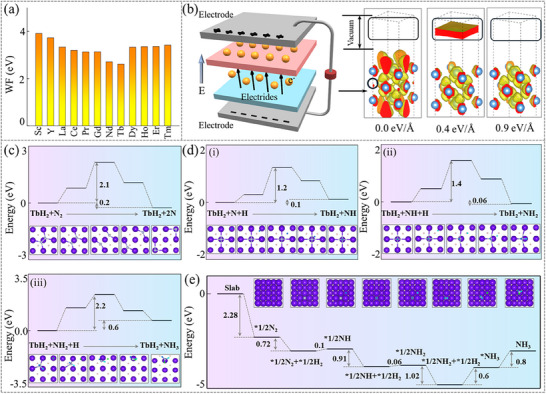
a) illustrates the WFs of twelve inorganic electrides. b) Left panel: Schematic diagram of the electronic emission device. Right panel: ELFs of CeH_2_ under different effective electric fields. c) shows energy profiles for N_2_ activation on the TbH_2_‐(001) surface. d) depicts energy profiles for (i) NH, (ii) NH_2_, and (iii) NH_3_ on the TbH_2_‐(001) surface. e) shows energy profiles of the reaction path for the NH_3_ synthesis on the TbH_2_‐(001) surface.

Inorganic electrides universally exhibit low WFs owing to the weakly bound nature of their IAEs. The calculations demonstrate that twelve 3D inorganic electrides display relatively low WFs along the [001] direction in their most stable magnetic ground states (see **Figure** **5**a; Figure S12, Supporting Information). Notably, the WFs of GdH_2_ (Φ_WF_ = 2.71 eV) and TbH_2_ (Φ_WF_ = 2.61 eV) are comparable to those of the first‐discovered inorganic electride C12A7 (Φ_WF_ = 2.4 eV),^[^ ^46^ ^]^ and 2D inorganic electride Ca_2_N (Φ_WF_ = 3.5 eV),^[^ ^47^ ^]^ highlighting their exceptional electron emission capabilities. Building upon this foundation, we have designed an electron emission device based on the following working principle (see Figure 5b): First, an inorganic electride‐target sample heterostructure is prepared by relevant experimental methods. Subsequently, under an applied electric field, 3D IAEs from the inorganic electride are injected into the target sample, achieving effective electron doping. Taking CeH_2_ (Φ_WF_ = 3.19 eV) as an example, one can find that when the applied electric field reaches 0.4 eV Å^−1^, the 3D IAEs are completely excited into the vacuum layer, forming a quasi‐2DEG. Furthermore, such a quasi‐2DEG vanishes upon increasing the field to 0.9 eV Å^−1^, suggesting the potential for realizing a switchable electron emission device based on the electric‐field‐induced transition (see Figure 5b).

Moreover, the low WF of inorganic electrides facilitates exceptional electron‐donating capability, enabling a large number of electrons to occupy the antibonding *π*‐orbital of N_2_. This unique characteristic renders them highly promising candidates for efficient NH_3_ synthesis catalysis. By selecting TbH_2_, which exhibits the lowest WF among them, we systematically investigated the reaction mechanism of 3D inorganic electrides in NH_3_ synthesis. As shown in Figure 5c, the 3D IAEs facilitate N_2_ activation (d_N≡N_ = 1.35 Å) on the TbH_2_‐(001) surface, with the dissociation energy barrier for N≡N bond cleavage into two N atoms being only 2.1 eV, comparable to that on the conventional Ru‐(001) surface (2.01 eV). Subsequently, NH_3_ synthesis simulations were performed using one of the dissociated N atoms as the reference site. Remarkably, the NH_3_ synthesis process on the TbH_2_‐(001) surface generates three distinct intermediate states: NH, NH_2_, and NH_3_. The successive hydrogenation steps to form NH, NH_2_, and NH_3_ intermediates need to overcome energy barriers of 1.2, 1.4, and 2.2 eV, respectively (see Figure 5d‐(i–iii)), which are very close to some results of inorganic electrides Er_5_Si_3_ (0.97, 1.26, 1.75 eV),^[^ ^31^ ^]^ Y_5_Si_3_ (0.81, 1.07, 1.64 eV),^[^ ^48^ ^]^ and LaRuSi (1.47, 1.61, 1.72 eV).^[^ ^49^ ^]^ Figure 5e shows the reaction path for the NH_3_ synthesis on the TbH_2_‐(001) surface. All the optimized configurations for NH_3_ synthesis are presented in Figure S13 (Supporting Information).

## 5 Conclusion 

In summary, based on geometric cavity design in crystal structures, we have identified twelve inorganic electrides with 3D IAEs, namely rare earth‐based hydrides ReH_2_ (Re = Sc, Y, La, Ce, Pr, Nd, Gd, Tb, Dy, Ho, Er, Tm). Among them, Sc/Y/LaH_2_, Ce/NdH_2_, and Pr/Gd/Tb/Dy/Ho/Er/TmH_2_ exhibited NM, FM, and AFM behaviors, respectively. Additionally, these 3D inorganic electrides exhibit a rich variety of topological states, including Weyl points with distinct dispersion characteristics, QTDPs, CWNLs, and OLWNLs, along with corresponding Fermi arc and drumhead‐like surface states. When SOC is considered, CeH_2_—a representative 3D inorganic electride—exhibits gap openings at band crossing points near the Fermi level due to the breaking of *T* by its FM ordering. This results in a pronounced anomalous Hall conductivity of 939 S cm^−1^ at 0.06 eV above the Fermi level, which can be tuned toward the Fermi level through electron doping. Remarkably, these inorganic electrides demonstrate exceptionally low WFs (ranging from 2.6 to 3.9 eV), facilitating the electric‐field‐driven transfer of 3D IAEs and enhanced catalytic performance in NH_3_ synthesis. These findings open new horizons for exploring 3D TIEs.

## 6 Calculation Methods

All crystal structure files used for the calculations were obtained from the Materials Project.^[^ ^50^ ^]^ To investigate the electronic, topological, surface, and transport properties of the ReH_2_ family, we performed first‐principles calculations based on the density functional theory (DFT), employing the Vienna ab initio Simulation Package.^[^ ^51^, ^52^, ^53^ ^]^ The exchange–correlation potential was treated using the generalized gradient approximation of the Perdew–Burke–Ernzerhof functional.^[^ ^54^ ^]^ The cutoff energy was set as 400 eV. The Brillouin zone of bulk and slab structures was sampled using a Monkhorst–Pack *k*‐mesh of 9 × 9 × 9 and 9 × 9 × 1, respectively. The energy convergence criterion for electronic self‐consistency was set as 10^−6^ eV. A vacuum spacing of 20 Å was introduced to avoid the interlayer interactions between the periodic images of the slab system. To properly describe the localized *d‐* and *f‐*orbitals, we employed the DFT+*U* approach. Specifically, a Hubbard *U* value of 2 eV was applied to the *d‐* orbitals of Sc, Y, and La atoms, while a *U* value of 6 eV was applied to the *f*‐orbitals of Ce, Pr, Nd, Gd, Tb, Dy, Ho, Er, and Tm. The magnetic ground state was calculated based on a unit cell using the above calculation parameters. The topological features of surface states were calculated based on the maximally localized Wannier functions,^[^ ^55^, ^56^ ^]^ realized by using the WANNIERTOOLS package and WANNIER90 code.^[^ ^57^, ^58^, ^59^, ^60^ ^]^ To accurately reproduce the DFT band structure, the *s*‐, *p*‐, *d*‐, and *f*‐orbitals of Ce atoms, along with the *s*‐orbitals of H atoms, were included in the Wannierization process. The Wannier‐Berry Python code was employed to map the Berry curvature onto the band structure.^[^ ^61^ ^]^ The intrinsic anomalous Hall conductivity was calculated on a dense k‐mesh of 151 × 151 × 151, using the Berry phase theory,^[^ ^62^ ^]^

(1) $$\sigma_{\alpha \beta }^{A}=-\frac{e^{2}}{\hbar V}\sum_{n,k}f_{nk}\Omega_{\alpha \beta }^{n}\left(k\right)$$

where *n*, **k**, and *f_nk_* are the band index, wave vector, and Fermi‐Dirac distribution function, respectively. The band‐ and momentum‐resolved Berry curvature is defined as

(2) Ωxynk=−∑n′≠n2Imψnkv^xψn′kψn′kv^yψnkεnk−εn′k2

where ${\hat{v}}_{x}$ and ${\hat{v}}_{y}$ are the velocity operators, and ψ_*n* **k**_ and ε_*n* **k**_ are the eigenstates and eigenvalues at band index *n* and momentum **k**. For the NH_3_ synthesis simulations, the TbH_2_ substrate was used in a 2 × 2 × 1 supercell structure. Remarkably, in all structural optimizations and transition state searches, only the surface‐exposed rare‐earth metal atom (Tb) and adsorbed atoms/molecules (H, N, N_2_, NH, NH_2_, NH_3_) were allowed to relax, while all other atoms (H and Tb) remained fixed. Besides, the calculation methods of adsorption energy: E_ad_ = E_slab+N2 –_ E_N2 –_ E_slab_, E_ad_ = E_slab+N –_ 1/2E_N2 –_ E_slab_, E_ad_ = E_slab+H2 –_ E_H2 –_ E_slab_, E_ad_ = E_slab+H –_ 1/2E_H2 –_ E_slab_, E_ad_ = E_slab+NH –_ E_slab+N‐H_, E_ad_ = E_slab+NH2 –_ E_slab+NH‐H_, and E_ad_ = E_slab+NH3 –_ E_slab+NH2‐H_.

## Conflict of Interest

The authors declare no conflict of interest.

## Supporting information



Supporting Information

## Data Availability

The data that support the findings of this study are available from the corresponding author upon reasonable request.
