# Supplementary material for: 3D Topological Inorganic Electrides: Screening, Properties, and Applications
Source: Adv Sci (Weinh). 2025 Jul 3;12(37):e07469. doi: 10.1002/advs.202507469 (PMC12499456; doi:10.1002/advs.202507469)
Supplement: Supplementary file 1 — Supporting Information [file ADVS-12-e07469-s001.docx]

*Supporting Information*

3D Topological Inorganic Electrides: Screening, Properties, and Applications

Zhenzhou Guo^#^, Weizhen Meng^#*^, Qianwen Zhang, Xia Cheng, Shiyao Wang, Yalong Jiao, Ying Liu^*^, Xiaoming Zhang, Zhenxiang Cheng, Tie Yang^*^

Table of Contents

[1. Topological inorganic electrides 2](#_Toc195989086)

[2. The electronegativity and magnetic ground state of the ReH_2_ family 3](#_Toc195989087)

[3. The atomic sites and lattice constants of the ReH_2_ family 6](#_Toc195989088)

[4. The magnetic ground state of CeH_2_ and the spin density of the ReH_2_ family 7](#_Toc195989089)

[5. Electron localization functions of the ReH_2_ family 8](#_Toc195989090)

[6. Part charge densities of the ReH_2_ family 9](#_Toc195989091)

[7. Electronic band structures of the ReH_2_ family 11](#_Toc195989092)

[8. The magnetic ground state and electronic structure of CeH_3_ 13](#_Toc195989093)

[9. Work functions of the ReH_2_ family 15](#_Toc195989094)

[10. Optimized configurations for NH_3_ synthesis in TbH_2_-(001) surface 16](#_Toc195989095)

**1. Topological inorganic electrides**

Bradlyn et al. demonstrated that electron distributions far beyond the atomic limit are critical for stabilizing topological states ^[1]^. Remarkably, the band inversion mediated by IAEs in electrides follows the same fundamental mechanism that drives topologically nontrivial band inversions in established topological materials. As shown in Fig. S1(a), when the conduction and valence bands arising from atomic orbital electrons form topological states near the Fermi level, the system is classified as a conventional topological material. However, only if interstitial anionic electrons are involved in forming topological states can the material be classified as a topological inorganic electride ^[1]^. Generally, topological inorganic electrides can be classified into three cases as depicted in Fig. S1(b-d).


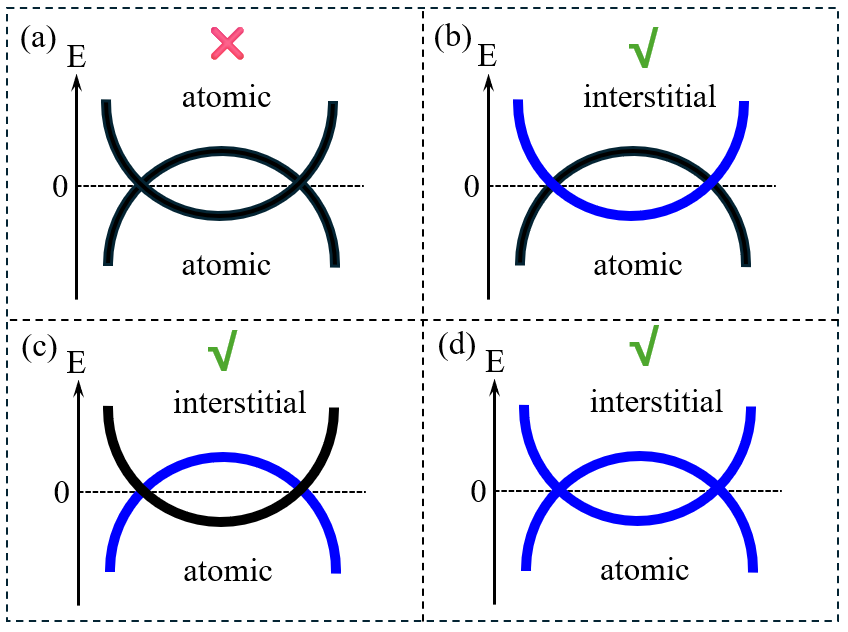


**Fig. S1. (a)** depicts the electronic band structure of a conventional topological material. **(b-d)** show the classification of electronic band structures in three distinct topological inorganic electrides.

**2. The electronegativity and magnetic ground state of the ReH_2_ family**

As shown in Table S1, all 12 rare-earth elements exhibit lower electronegativity than hydrogen, indicating that Re atoms in the ReH_2_ family are more prone to electron loss compared to H atoms. Consequently, Re and H atoms present positive and negative valence states, respectively. Table S2 and Fig. S2 show the energy values and magnetic moments of 9 3D inorganic electrides ReH_2_ under different magnetic configurations. Remarkably, previous studies have demonstrated that the ReH_2_ family can be synthesized ^[2-9]^. Taking CeH_2_ as an example, Ce chips (Purity: 99.9%) are placed in an argon-filled glovebox (O_2_, H_2_O < 1 ppm). Subsequently, Ce chips were heated at 400 °C under approximately 1 MPa of hydrogen for 5 hours, successfully yielding the CeH_2_ compound ^[2]^.

**Table S1.** The electronegativity of 12 rare earth (Re) elements and hydrogen (H) atom.

| Elements | Electronegativity | Elements | Electronegativity | Elements | Electronegativity |
| --- | --- | --- | --- | --- | --- |
| H | 2.20 | Sc | 1.36 | Y | 1.22 |
| La | 1.10 | Ce | 1.12 | Pr | 1.13 |
| Nd | 1.14 | Gd | 1.20 | Tb | 1.10 |
| Dy | 1.22 | Ho | 1.23 | Er | 1.24 |
| Tm | 1.25 |  |  |  |  |


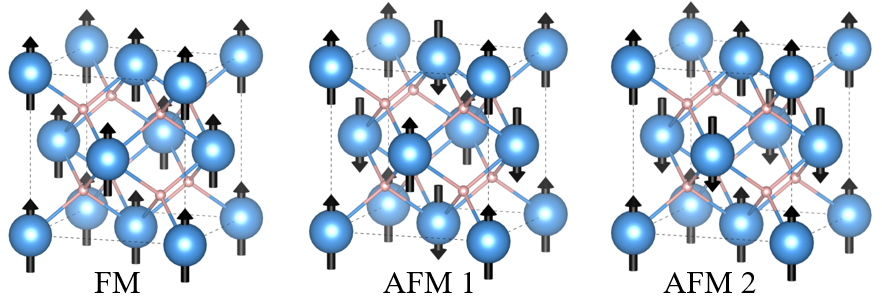


**Fig. S2.** shows three magnetic configurations of 9 3D magnetic inorganic electrides ReH_2_.

**Table S2.** Magnetic ground states and magnetic moments of 9 3D magnetic inorganic electrides ReH_2_ (Re= Ce, Pr, Nd, Gd, Tb, Dy, Ho, Er, Tm).

| System-CeH_2_ (MS-NM) | Energy (eV) | Magnetic moment (μ_B_/Ce atom) |
| --- | --- | --- |
| AFM1 | -47.778745 | 0.698 |
| AFM2 | -47.778748 | 0.698 |
| FM | -48.094885 | 0.698 |
| NM | -47.916530 | 0 |

| System-PrH_2_ (MS-NM) | Energy (eV) | Magnetic moment (μ_B_/Pr atom) |
| --- | --- | --- |
| AFM1 | -58.603407 | 2.023 |
| AFM2 | -58.604640 | 2.023 |
| FM | -53.191641 | 2.562 |
| NM | -39.502066 | 0 |

| System-NdH_2_ (MS-NM) | Energy (eV) | Magnetic moment (μ_B_/Nd atom) |
| --- | --- | --- |
| AFM1 | -63.571586 | 3.024 |
| AFM2 | -63.571589 | 3.024 |
| FM | -63.589321 | 3.049 |
| NM | -38.124898 | 0 |

| System-GdH_2_ (MS-FM) | Energy (eV) | Magnetic moment (μ_B_/Gd atom) |
| --- | --- | --- |
| AFM1 | -.90.676089 | 7.108 |
| AFM2 | -90.676079 | 7.108 |
| FM | -90.675728 | 7.108 |
| NM | -47.393899 | 0 |

| System-TbH_2_ (MS-NM) | Energy (eV) | Magnetic moment (μ_B_/Tb atom) |
| --- | --- | --- |
| AFM1 | -74.281624 | 6.085 |
| AFM2 | -74.281628 | 6.085 |
| FM | -74.272897 | 6.083 |
| NM | -42.968133 | 0 |

| System-DyH_2_ (MS-NM) | Energy (eV) | Magnetic moment (μ_B_/Dy atom) |
| --- | --- | --- |
| AFM1 | -71.934451 | 4.447 |
| AFM3 | -71.934201 | 4.447 |
| FM | -68.506092 | 4.871 |
| NM | -42.481109 | 0 |

| System-HoH_2_ (MS-NM) | Energy (eV) | Magnetic moment (μ_B_/Ho atom) |
| --- | --- | --- |
| AFM1 | -70.813259 | 4.062 |
| AFM2 | -70.813262 | 4.062 |
| FM | -67.798593 | 3.178 |
| NM | -46.334755 | 0 |

| System-ErH_2_ (MS-NM) | Energy (eV) | Magnetic moment (μ_B_/Er atom) |
| --- | --- | --- |
| AFM1 | -64.438147 | 3.000 |
| AFM2 | -.64.438156 | 3.000 |
| FM | -64.181537 | 3.027 |
| NM | -52.580514 | 0 |

| System-TmH_2_ (MS-NM) | Energy (eV) | Magnetic moment (μ_B_/Tm atom) |
| --- | --- | --- |
| AFM1 | -57.086532 | 1.287 |
| AFM2 | -57.086535 | 1.287 |
| FM | -51.876040 | 1.582 |
| NM | -52.120517 | 0 |

| Chemical formula | Magnetism | Magnetic moment (μ_B_) | Chemical formula | Magnetism | Magnetic moment (μ_B_) |
| --- | --- | --- | --- | --- | --- |
| CeH_2_ | FM | 0.698 | DyH_2_ | AFM | 4.447 |
| PrH_2_ | AFM | 2.023 | HoH_2_ | AFM | 4.062 |
| NdH_2_ | FM | 3.049 | ErH_2_ | AFM | 3.000 |
| GdH_2_ | AFM | 7.108 | TmH_2_ | AFM | 1.287 |
| TbH_2_ | AFM | 6.085 | YH_2_ | NM | 0.000 |
| ScH_2_ | NM | 0.000 | LaH_2_ | NM | 0.000 |

**3. The atomic sites and lattice constants of the ReH_2_ family**

The ReH_2_ family belongs to the cubic *Fm-3m* structure (SG 225), where the rare-earth atom occupies the (0, 0, 0) site and two hydrogen atoms occupy the (0.25, 0.25, 0.25) and (0.75, 0.75, 0.75) sites, as shown in Table S3. Table S4 shows the lattice constants of 12 3D inorganic electrides.

**Table S3.** Space group and Wyckoff positions of atoms in ReH_2_ family.

| Chemical formula | Space group | Unit cell parameters | |
| --- | --- | --- | --- |
| ReH_2_ | *Fm-3m* (No. 225) | 𝛼, 𝛽, 𝛾(°) | |
|  |  | 60, 60, 60 | |
| Sites | Wyckoff positions | Coordinates ($\frac{x}{a}$, $\frac{y}{b}$ , $\frac{z}{c}$) | Site symmetry |
| Re | 1a | (0.0, 0.0, 0.0) | 1 |
| H_1_ | 1a | (0.25, 0.25, 0.25) | 1 |
| H_2_ | 1a | (0.75, 0.75, 0.75) | 1 |

**Table S4.** The lattice constants of ReH_2_ family.

| Chemical formula | Lattice constant (Å) | Chemical formula | Lattice constant (Å) |
| --- | --- | --- | --- |
| ScH_2_ | 3.37161 | LaH_2_ | 3.99964 |
| YH_2_ | 3.67510 | CeH_2_ | 3.89372 |
| PrH_2_ | 3.926848 | GdH_2_ | 3.739443 |
| NdH_2_ | 3.880788 | TbH_2_ | 3.691455 |
| DyH_2_ | 3.662188 | ErH_2_ | 3.603599 |
| HoH_2_ | 3.633995 | TmH_2_ | 3.576899 |

**4. The magnetic ground state of CeH_2_ and the spin density of the ReH_2_ family**


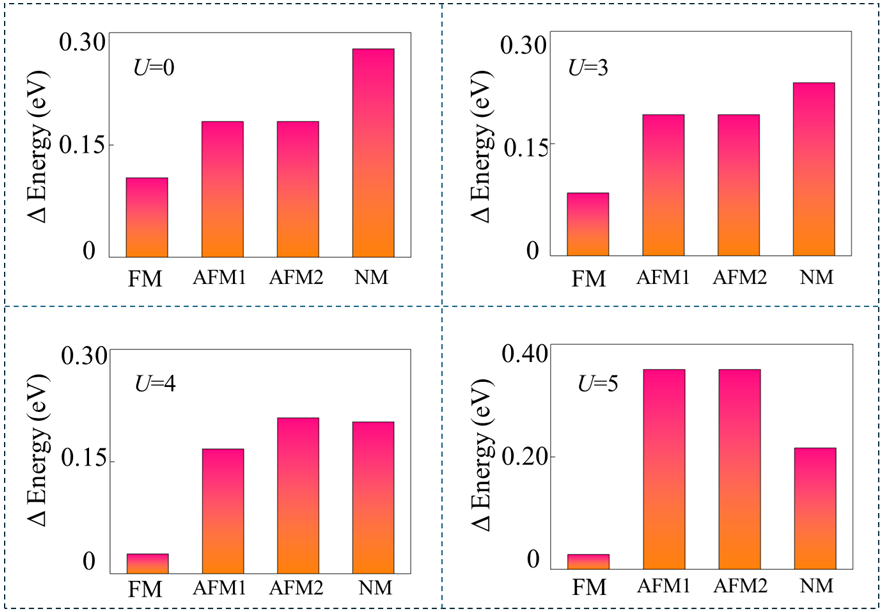


**Fig. S3.** The energy values corresponding to different magnetic configurations under different *U* values in CeH_2_.


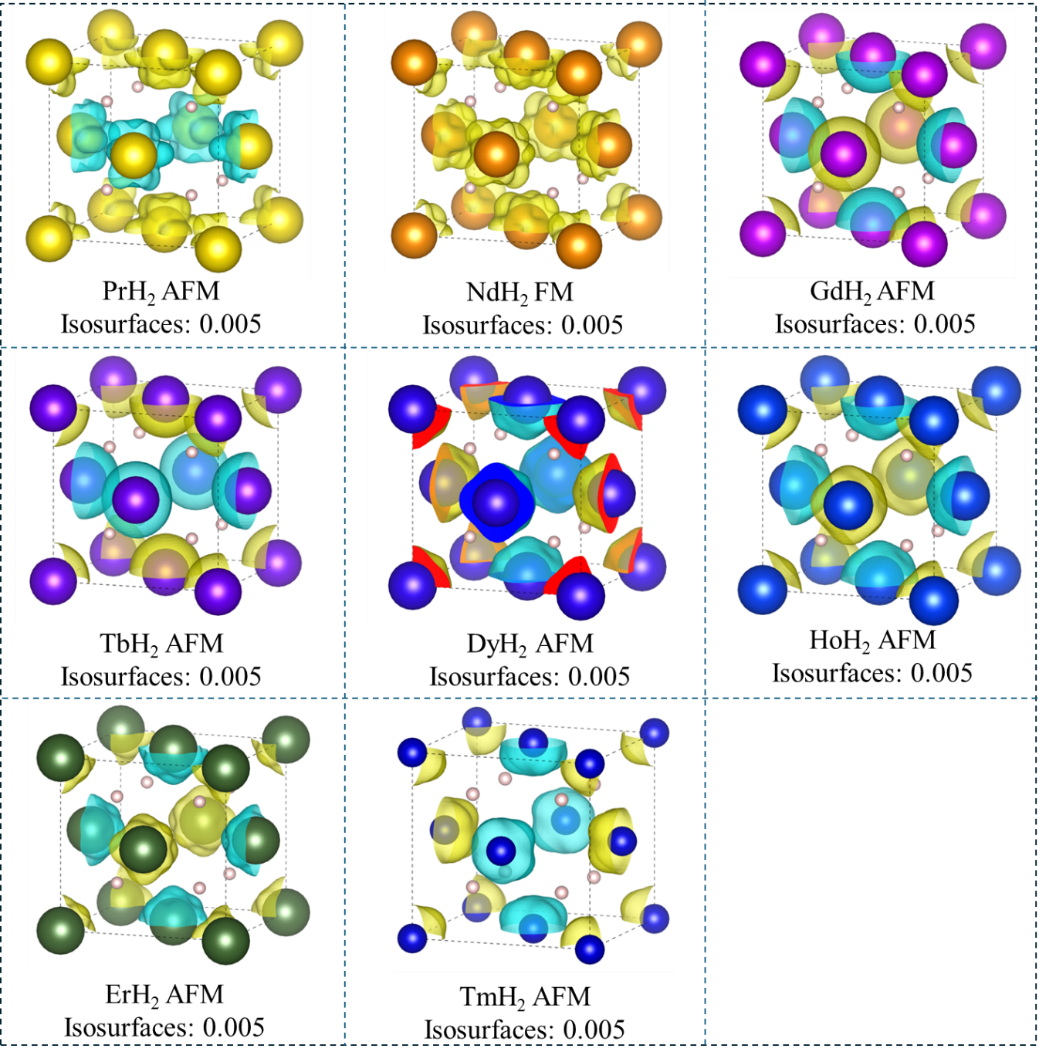


**Fig. S4.** The spin density of ReH_2_ family.

**5. Electron localization functions of the ReH_2_ family**


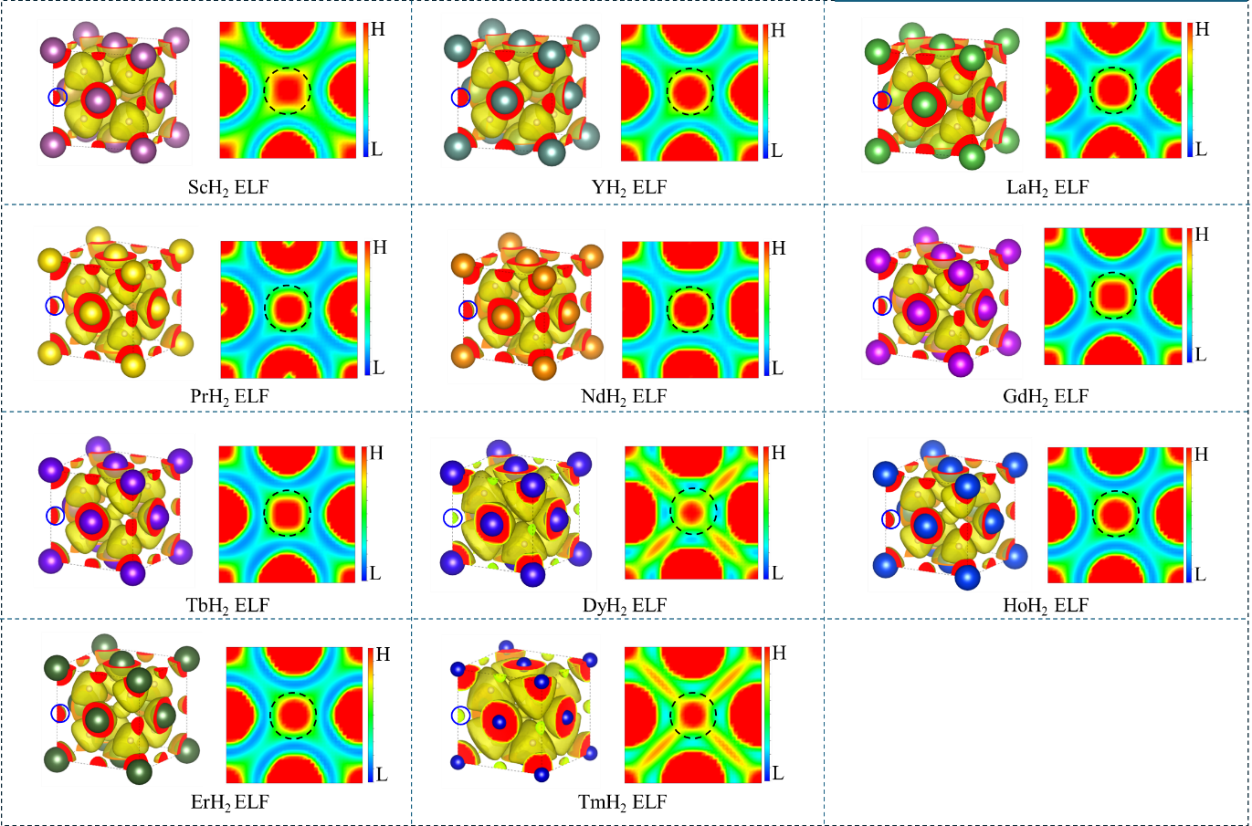


**Fig. S5.** Electron localization functions of the ReH_2_ family.

**6. Part charge densities of the ReH_2_ family**


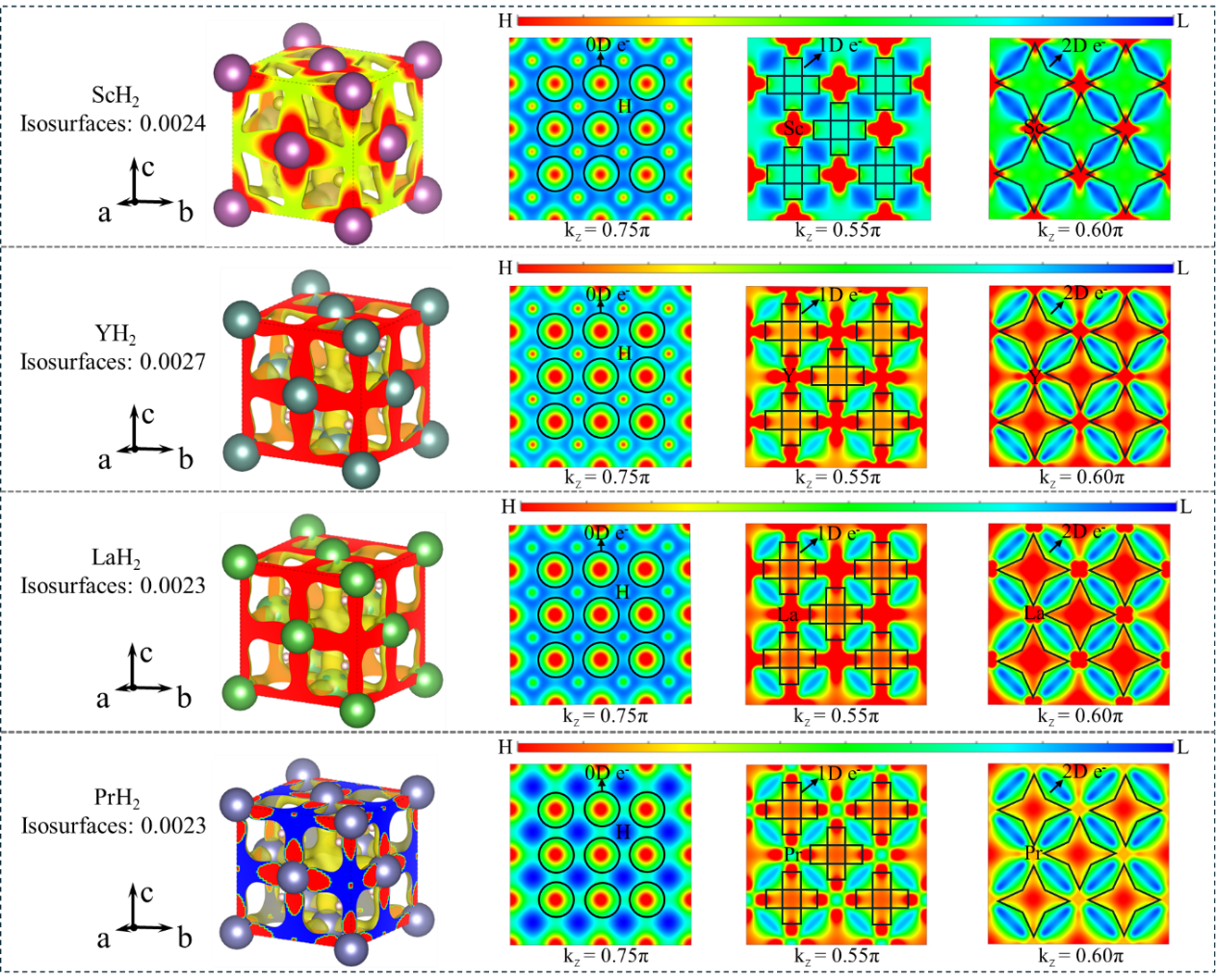


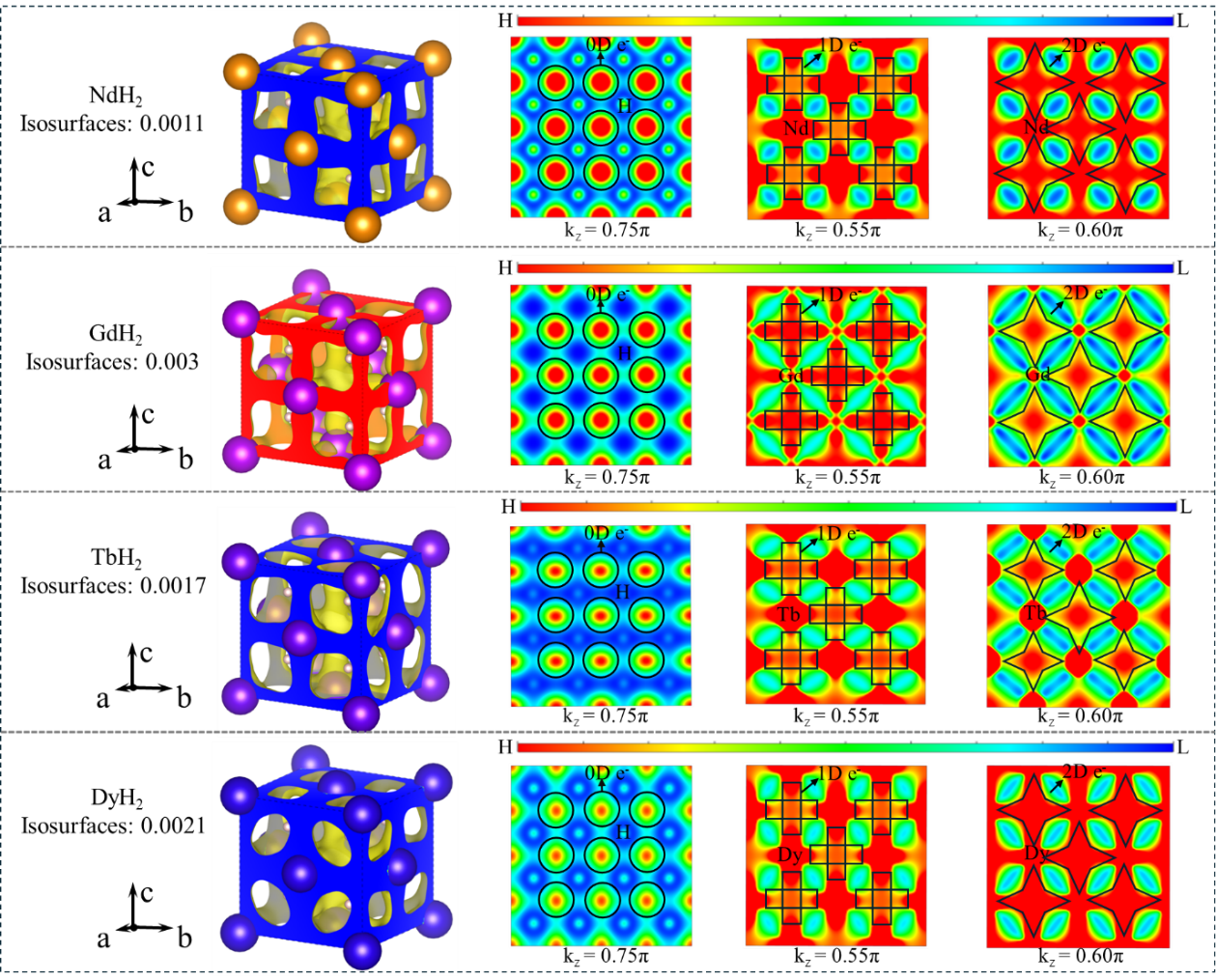


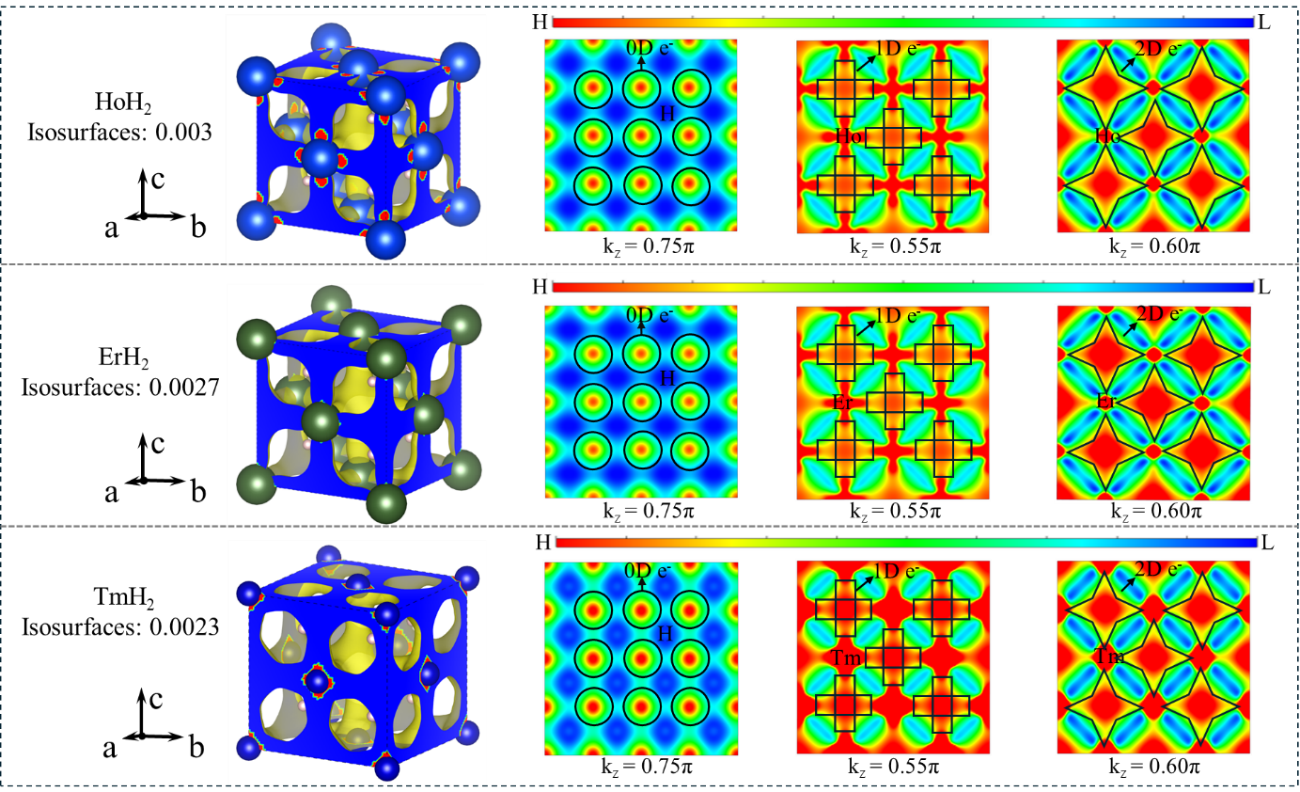


**Fig. S6.** Part charge densities of the ReH_2_ family.

**7. Electronic band structures of the ReH_2_ family**

In addition to the 3D FM topological inorganic electride CeH_2_ mentioned in the main text, the other candidates also demonstrate a rich variety of topological states, such as critical Weyl point (CWP), quadratic Weyl point (QWP), linear Weyl point (LWP), quadratic triple degenerate point (QTDP), quadratic nodal line (QNL), and linear nodal line (LNL), *etc*., as shown in Fig. S7. Remarkably, the surface states of QTDPs show 1D Fermi arcs, whereas QNLs display 2D drumhead-like surface states. This fundamental difference implies that the surface state density of NLs is substantially higher than that of TDPs. Besides, linear and quadratic TDPs/NLs are different in their slopes of band dispersions, as shown in Fig. S8.


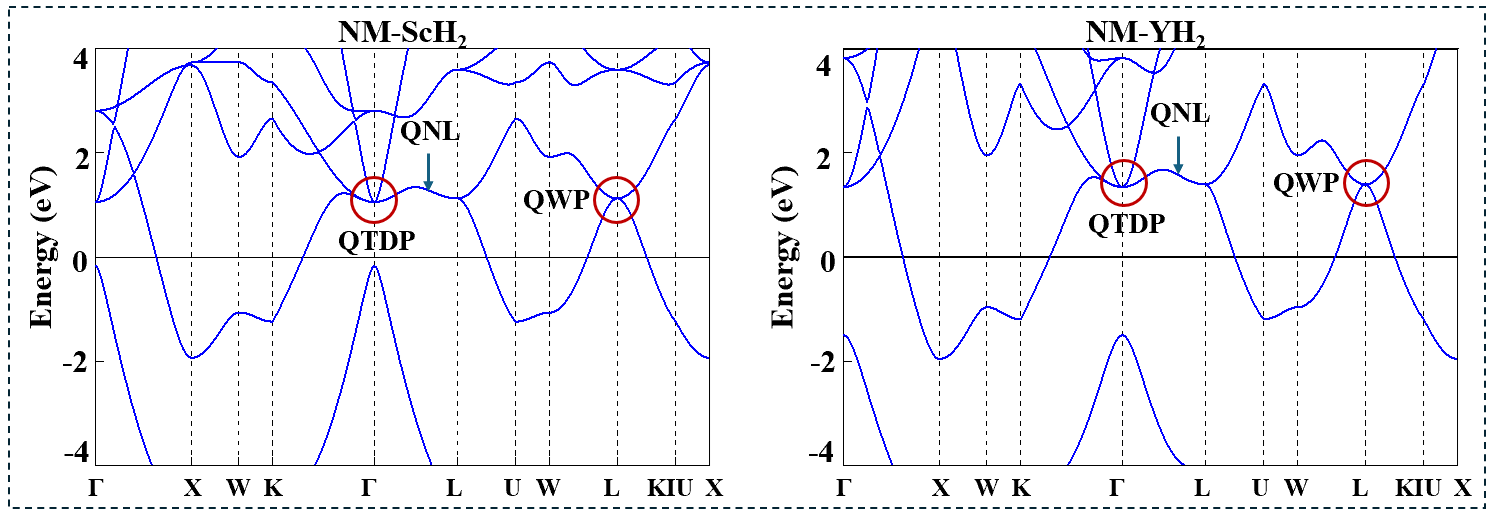


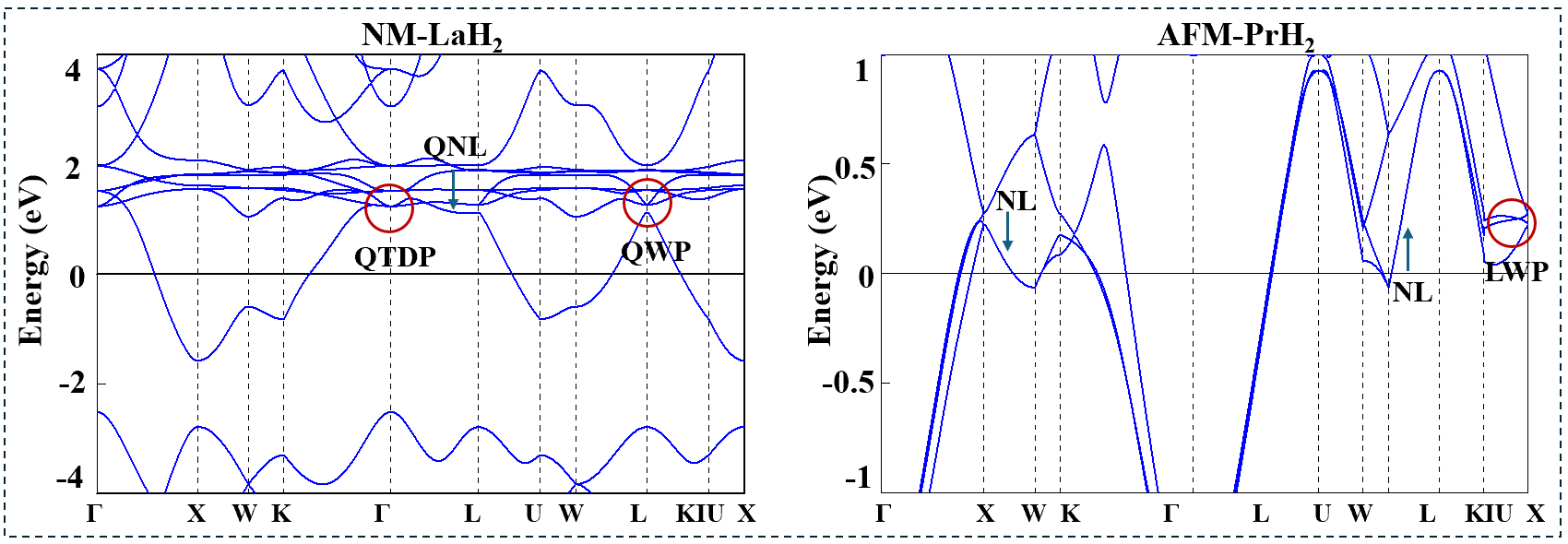


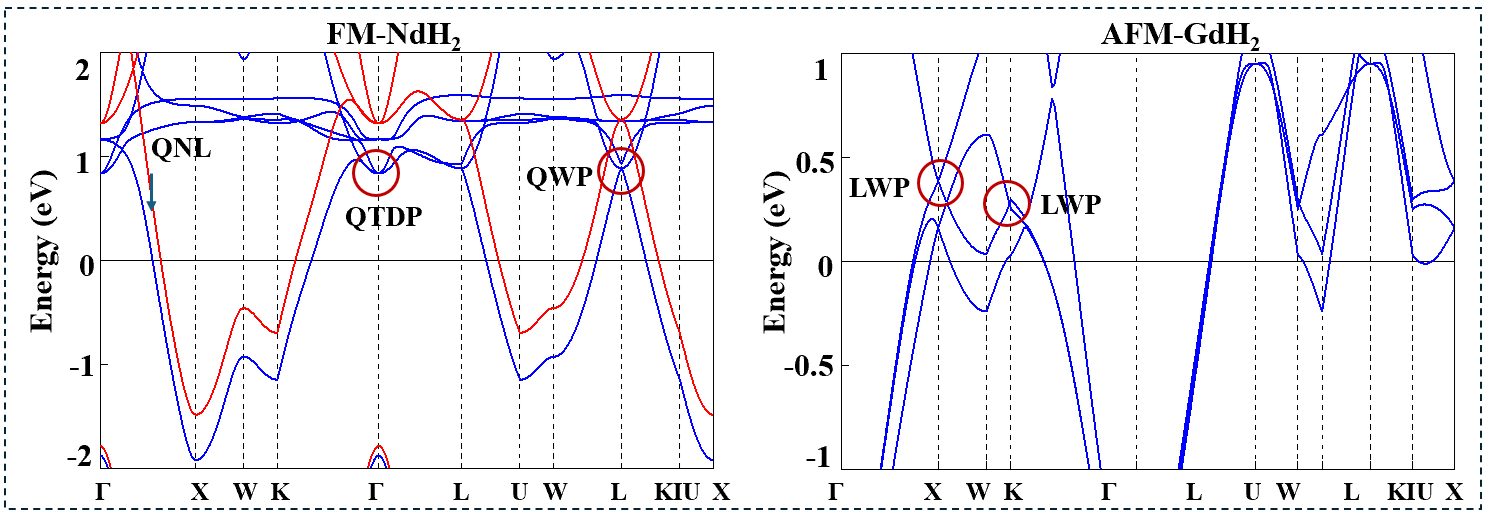


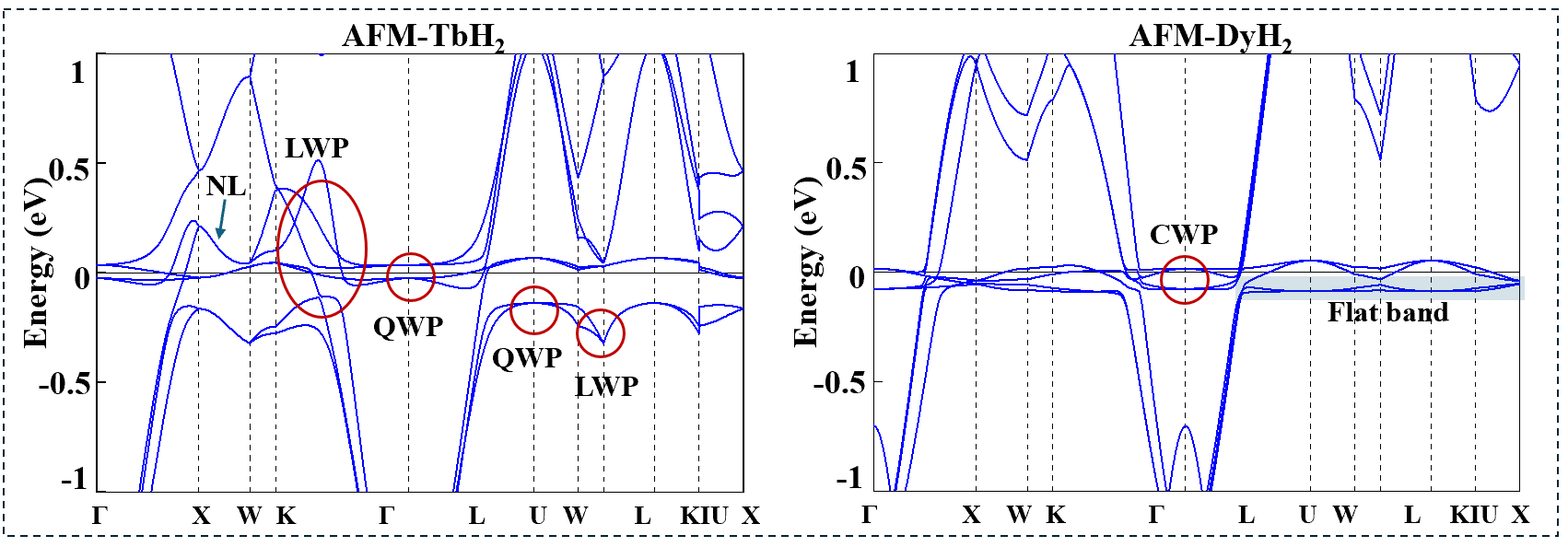


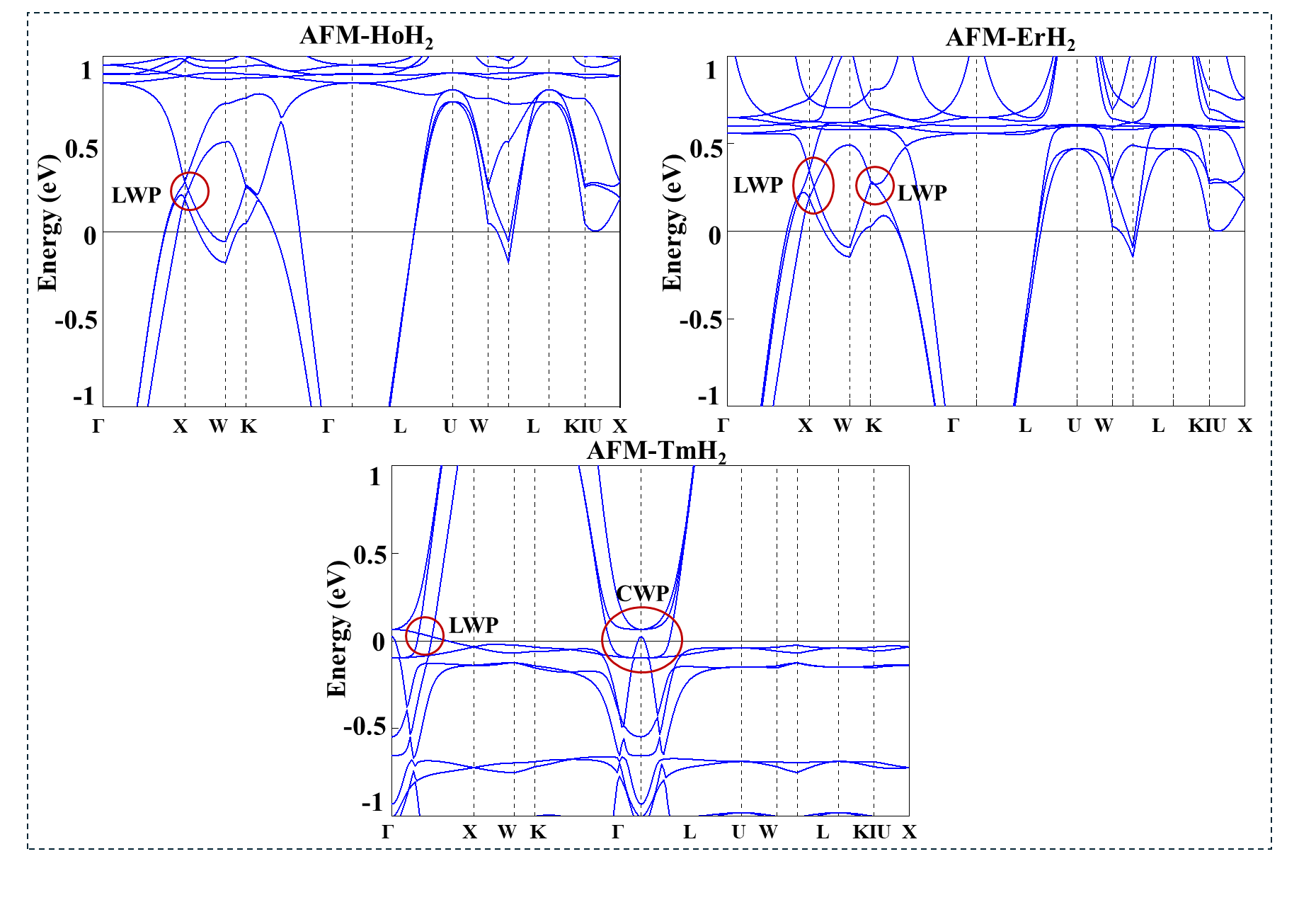


**Fig. S7**. The electronic band structures of ReH_2_ family.


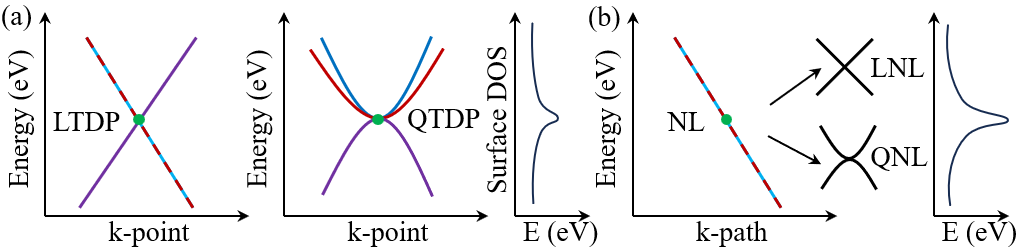


**Fig. S8.** Schematic figures for LTDP, QTDP, LNL, and QNL: (a) shows the band structures and surface DOS of LTDP and QTDP. (b) shows the band structures and surface DOS of LNL and QNL.

**8. The magnetic ground state and electronic structure of CeH_3_**


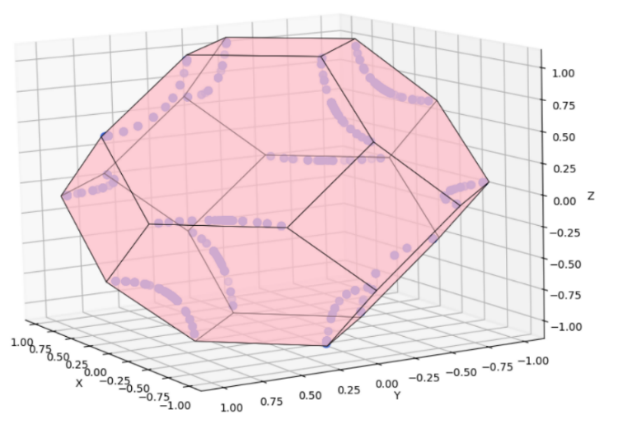


**Fig. S9.** shows the nodal loop around point K in CeH_2_.

The calculations demonstrate that CeH_3_ still maintains the FM ground state with the [100] easy magnetization axis, which agrees well with the result of the experiment ^[10]^, as shown in Fig. S10.


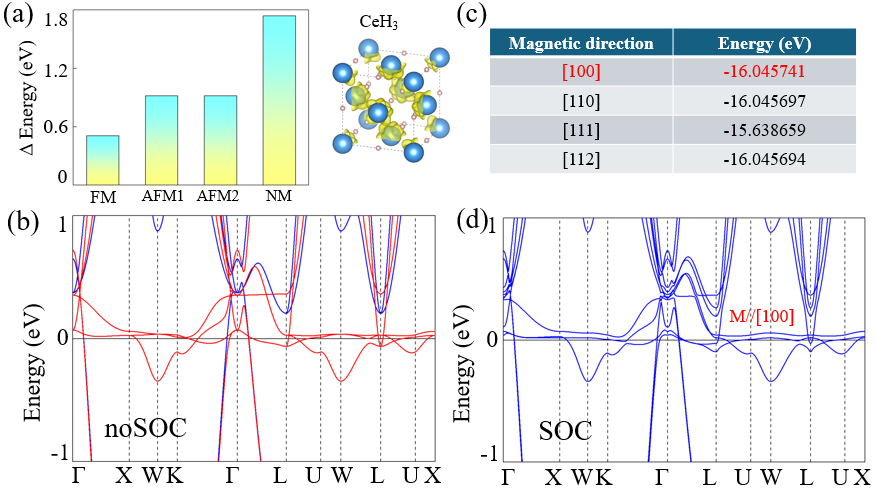


**Fig. S10. (a)** shows the energies of different magnetic configurations and the spin density map of FM CeH_3_. **(b)** shows the electronic band structure without SOC for CeH_3_. **(c)** shows the magnetic anisotropy energy under different crystallographic directions for CeH_3_. **(d)** shows the electronic band with SOC along the [100] direction.


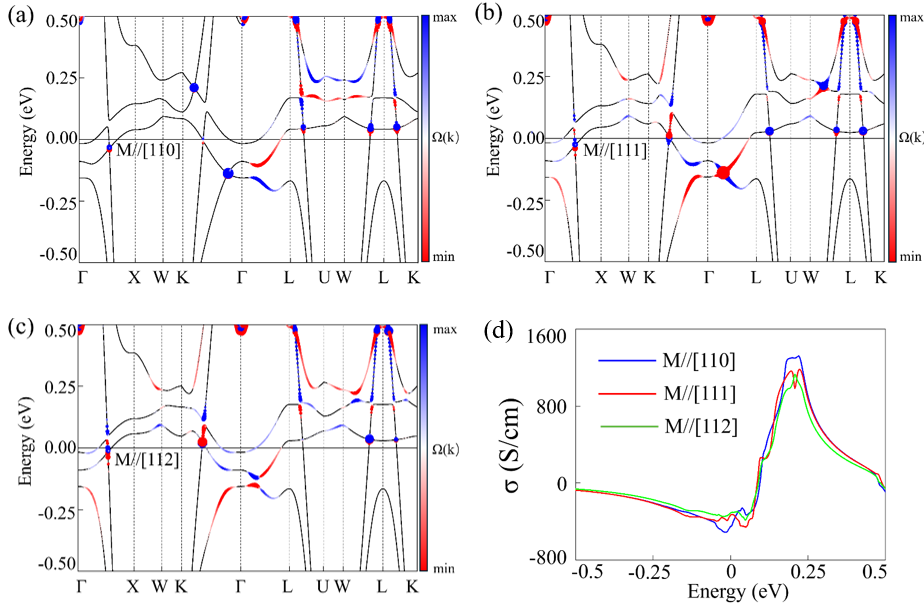


**Fig. S11.** The projected Berry curvature distribution on the electronic bands of CeH_2_ with the magnetization moment along (a) [110], (b) [111] and (c) [112] directions, respectively. (d) The energy dependence of AHC of CeH_2_ with the magnetization moment along the [110], [111] and [112] directions, respectively.

**9. Work functions of the ReH_2_ family**


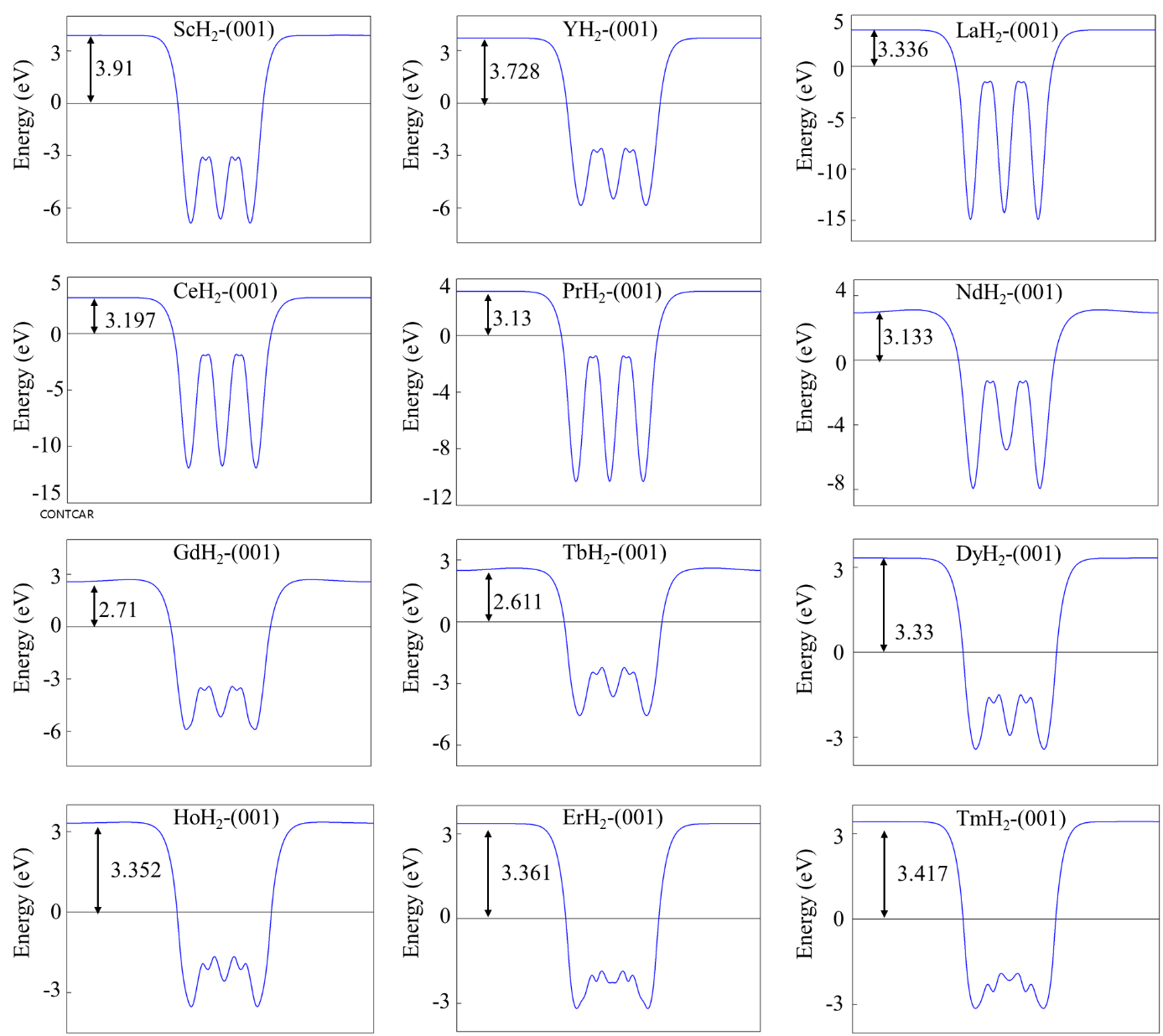


**Fig. S12.** shows the work functions of the ReH_2_ family.

| Chemical formula | WFs (eV) | Chemical formula | WFs (eV) |
| --- | --- | --- | --- |
| CeH_2_ | 3.19 | DyH_2_ | 3.33 |
| PrH_2_ | 3.13 | HoH_2_ | 3.35 |
| NdH_2_ | 3.13 | ErH_2_ | 3.36 |
| GdH_2_ | 2.71 | TmH_2_ | 3.41 |
| TbH_2_ | 2.61 | YH_2_ | 3.71 |
| ScH_2_ | 3.91 | LaH_2_ | 3.33 |

**10. Optimized configurations for NH_3_ synthesis in TbH_2_-(001) surface**

When inorganic electrides are loaded with transition metal Ru, two major challenges may arise during experimental preparation. First, the lattice mismatch between some inorganic electrides and transition metal Ru makes it inherently difficult to deposit Ru nanoparticles on these substrates. Second, Ru nanoparticles tend to undergo severe aggregation when grown on the surface of inorganic electrides, significantly reducing their surface activity. Consequently, exploring freestanding inorganic electrides for NH_3_ synthesis is critically necessary. Previous studies primarily focused on intermetallic electrides such as LaScSi ^[12]^. Our recent work reveals that rare-earth-based inorganic electrides may serve as potential candidates, with the ReH_2_ family representing a quintessential example.


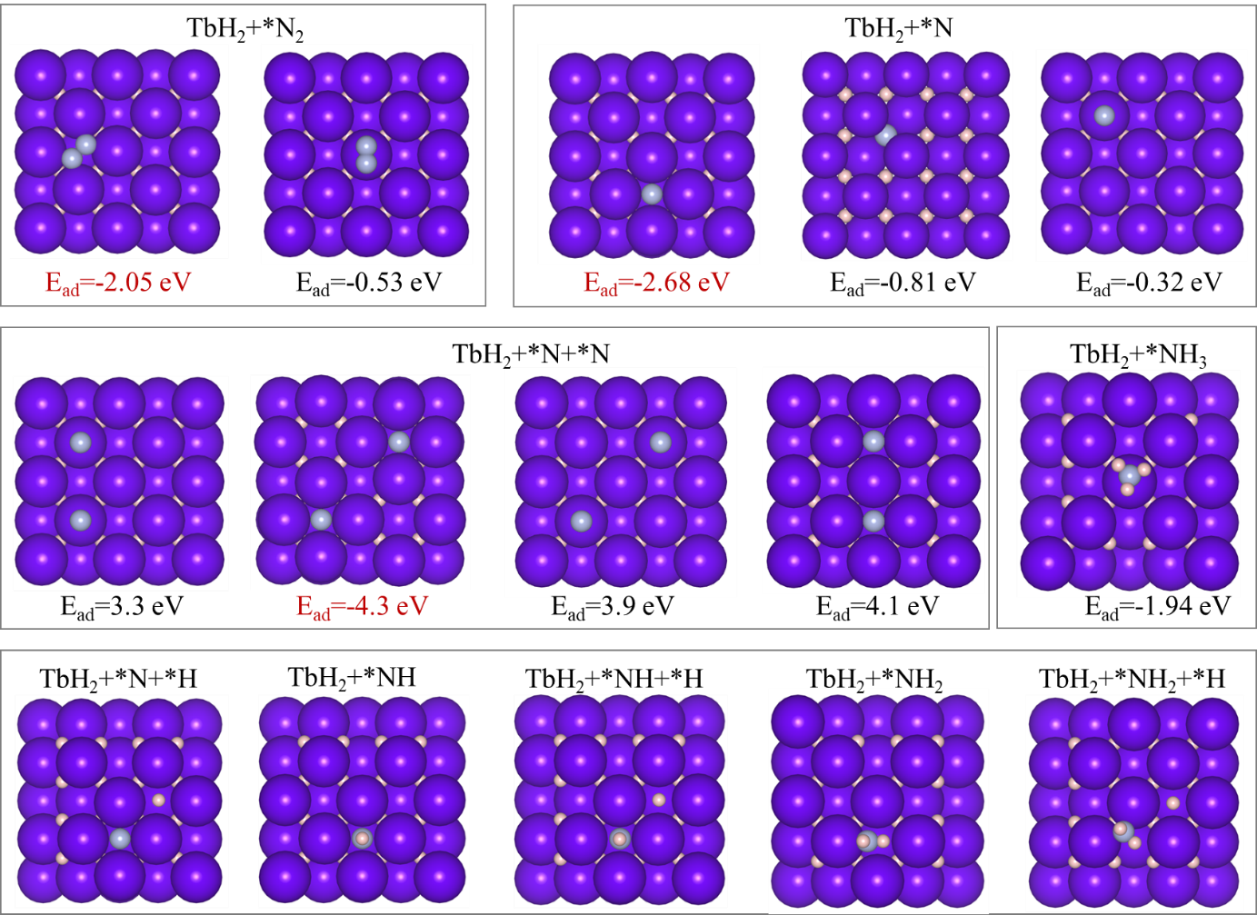


**Fig. S13.** shows the optimized configurations of N, N+N, N_2_, N+H, NH, NH+H, NH_2_, NH_2_+H, and NH_3_ on the TbH_2_-(001) surface.

**References**

[S1] M. Hirayama, R. Takahashi, S. Matsuishi, H. Hosono, S. Murakami, *Phys. Rev. Research* **2020**, 2, 043131.

[S2] H. Mizoguchi, M. Okunaka, M. Kitano, S. Matsuishi, T. Yokoyama, H. Hosono, *Inorg. Chem.* **2016**, *55*, 8833.

[S3] T. Kobayashi, H. Takei, *J. Cryst. Growth* **1978**, *45*, 29.

[S4] W. L. Korst, J. C. Warf, *Inorg. Chem.* **1966**, *5*, 1719.

[S5] S. Hémon, R. A. Cowley, R. C. C. Ward, M. R. Wells, L. Douysset, H. Ronnow, *J. Phys.: Condens. Matter* **2000**, *12*, 5011.

[S6] Y.-L. Hai, N. Lu, H.-L. Tian, M.-J. Jiang, W. Yang, W.-J. Li, X.-W. Yan, C. Zhang, X.-J. Chen, G.-H. Zhong, *J. Phys. Chem. C* **2021**, *125*, 3640.

[S7] P. Vajda, J. N. Daou, *Phys. Rev. B* **1992**, *45*, 9749.

[S8] J. Dudáš, S. Gabáni, V. Kavečanský, I. Gościańska, J. Bagi, *Acta Phys. Pol. A* **2010**, *118*, 843.

[S9] J. A. Grimshaw, F. J. Spooner, C. G. Wilson, A. D. McQuillan, *J. Mater. Sci.* **1981**, *16*, 2855.

[S10] D. Shaltiel, H. Winter, E. Dormann, J. P. Burger, J. N. Daou, P. Vajda, A. Grayevsky, *J. Less-Common Met.* **1991**, *172–174*, 293.

[S11] J. Osterwalder, H. R. Ott, L. Schlapbach, J. Schefer, P. Fischer, *Journal of the Less Common Metals* **1983**, 94, 1, 129-137.

[S12] W. Meng, L. Tian, F. Zhou, Z. Mo, Y. Jiao, S. Wang, J. Jiang, X. Zhang, Z. Cheng, Y. Liu, W. Wang, G. Zhang, X. Wang, *Advanced Materials* **2025**, *37*, 2418904.
